# Impact of Virtual Reality–Based Biofeedback on Sleep Quality Among Individuals With Depressive Symptoms, Anxiety Symptoms, or Both: 4-Week Randomized Controlled Study

**DOI:** 10.2196/65772

**Published:** 2025-06-20

**Authors:** Sisu Seong, Hyewon Kim, Yaehee Cho, Min-Ji Kim, Ka Ram Park, Jooeun Choi, Seonah Lee, Dong Jun Kim, Seog Ju Kim, Hong Jin Jeon

**Affiliations:** 1 Department of Medical Device Management and Research, Samsung Advanced Institute for Health Sciences & Technology, Sungkyunkwan University, Seoul, Republic of Korea; 2 Department of Psychiatry, Hallym University Sacred Heart Hospital, Anyang, Republic of Korea; 3 Department of Psychiatry, Depression Center, Samsung Medical Center, Sungkyunkwan University School of Medicine, 81, Irwon-ro, Gangnam-gu, Seoul, 06351, Republic of Korea, 821034103586; 4 Biomedical Statistics Center, Research Institute for Future Medicine, Samsung Medical Center, Seoul, Republic of Korea; 5 Meditrix Co., Ltd., Seoul, Republic of Korea; 6 Department of Health Sciences & Technology and Department of Clinical Research Design & Evaluation, Samsung Advanced Institute for Health Sciences & Technology, Sungkyunkwan University, Seoul, Republic of Korea

**Keywords:** virtual reality, biofeedback, PSQI, sleep quality, depression, Pittsburgh Sleep Quality Index

## Abstract

**Background:**

Use of virtual reality (VR)–based biofeedback (BF) represents an emerging nonpharmacological intervention for enhancing sleep quality in individuals exhibiting depressive symptoms, anxiety symptoms, or both. However, empirical evidence regarding its efficacy in addressing sleep disturbances remains limited and inconclusive.

**Objective:**

This 3-arm randomized controlled trial aimed (1) to compare the efficacy of VR-based BF with conventional BF in improving sleep quality, as measured by the Pittsburgh Sleep Quality Index (PSQI), among individuals with depressive symptoms, anxiety symptoms, or both (DAS); (2) to examine the effects of VR-based BF in a demographically similar healthy control (HC) group; and (3) to evaluate between-group differences in sleep quality improvements at the 4-week follow-up.

**Methods:**

Participants scoring ≥10 on the Patient Health Questionnaire-9 or ≥9 on the Panic Disorder Severity Scale were allocated to a group with DAS while others were assigned to a HC group. The DAS group was subsequently randomized into VR-based BF or conventional BF interventions with a therapist. All participants attended sessions at weeks 0, 2, and 4, completing assessments including the Montgomery-Asberg Depression Rating Scale, State-Trait Anxiety Inventory, and Visual Analog Scale in interviews. The PSQI was administered at baseline and postintervention to evaluate alterations in sleep quality over a 4-week period.

**Results:**

A total of 118 participants were randomized into a VR-based BF group (DAS/VR, n=40) or a conventional BF group (DAS/BF, n=38), and a control group (HC/VR, n=40) received VR-based BF. Sleep disturbance scores of both DAS/VR and DAS/BF groups had significant improvements (mean reductions of −0.58, SD 0.75 and −0.66, SD 0.75, respectively) compared to those preintervention, showing no significant difference after adjusting for age and sex (*P*=.49). The DAS/VR group had a greater improvement in sleep disturbance (mean −0.08, SD 0.53; *P*=0.0014) than the HC/VR group. Global PSQI scores in both DAS/VR and DAS/BF groups improved compared to those preintervention, showing decreases by −2.50 (SD 2.89) and −3.39 (SD 2.80), respectively. The difference between the 2 groups was not statistically significant (*P*=.14). The Global PSQI score in the DAS/VR group showed significant improvement (−0.95, SD 2.09; *P*=.01) compared to that in the HC/VR group.

**Conclusions:**

This study provides evidence that both VR-based BF and conventional BF with a therapist are efficacious psychological interventions for enhancing sleep quality in individuals with depressive symptoms, anxiety symptoms, or both, with no significant differences observed between these 2 approaches. Both interventions showed significant improvements compared to baseline measurements. These findings suggest potential applications of these interventions in clinical settings to improve sleep quality and mental well-being.

## Introduction

In the United States, 84.7% of individuals with major depressive disorder (MDD) have reported sleep difficulties associated with higher depression severity scores [1]. Previous studies have demonstrated that poor sleep can significantly affect mental health and daily functioning, linking it to worsened mental health and reduced socio-occupational performance with serious mental illness [2-4]. Poor sleep in children and adolescents is likely to lead to mood disorders, emotional dysregulation, and increased risk of anxiety and suicidal thoughts [5]. Good sleep quality can enhance resilience and reduce depressive and anxiety symptoms, even in those without clinical conditions [6]. These findings underscore the importance of prioritizing sleep quality as a therapeutic target, particularly in interventions for individuals with depressive symptoms.

Biofeedback (BF) therapy helps individuals regulate physiological processes using real-time feedback on heart rate, respiration, and skin conductance [7]. BF uses paced breathing to boost vagal activity, balance the autonomic system, promote relaxation, and improve sleep quality [8]. BF can alleviate hypervigilance, anxiety, and depression by enhancing parasympathetic activity [9]. It can improve sleep latency, efficacy, and overall quality [10].

However, there are several specific obstacles associated with BF. Maintaining attentiveness during BF sessions present a significant challenge, as conventional methodologies often fail to adequately engage participants, potentially diminishing efficacy of the intervention [11,12]. There are inconsistencies in reported efficacies of BF across different domains and populations. While some studies have shown significant improvements in various aspects such as executive functions, stress reduction, and symptom management, others have reported mixed or limited results [9,13,14]. Such variability in outcomes suggests that BF might be more efficacious for certain populations or specific conditions. Further research is needed to establish its relevance and applicability. The lack of standard, controlled protocols and consistent reporting of efficacy sizes might hinder the establishment of BF as a widely accepted therapeutic approach [13].

Virtual reality (VR) operates by deflecting attention and submerging users in soothing simulations designed to activate and regulate different physiological and mental functions that may induce sleep [15]. However, research gaps remain in long-term effects and real-life generalizability of VR-based BF, despite observed short-term benefits [11,16]. Current research studies lack sample diversity. They focus mainly on healthy adults. More studies on clinical populations, children, and adolescents are needed [11,12,17]. This study addresses these gaps by evaluating the efficacy of VR-based BF on sleep quality in individuals with depressive symptoms, anxiety symptoms, or both using the Pittsburgh Sleep Quality Index (PSQI) [18] as a validated outcome measure and incorporating a diverse group structure to provide actionable insights.

Our hypothesis posited that VR-based BF would demonstrate superior efficacy in enhancing sleep quality compared to conventional BF among individuals with depressive symptoms, anxiety symptoms, or both (DAS) group, while also showing measurable effects in healthy control (HC) group. The primary objective of this 3-arm randomized controlled trial (RCT) was to conduct a head-to-head comparison of sleep quality improvements between (1) DAS/VR group (VR-based BF), (2) DAS/BF group (conventional BF), and (3) HC/VR group (VR-based BF) over a 4-week intervention period.

## Methods

### Participants

The screening process involved the use of the Korean version of the Mini International Neuropsychiatric Interview [19] to comply with the *Diagnostic and Statistical Manual of Mental Disorders, Fifth Edition* (*DSM-*5). This study had 2 groups: (1) DAS and (2) HC group. Inclusion criteria for the DAS group were as follows: those with self-reported subjective depression or anxiety, those who had no psychiatric treatment within the past 6 months, and those who scored≥10 on the Patient Health Questionnaire-9 (PHQ-9) [20] or ≥9 on the Panic Disorder Severity Scale (PDSS) [21].

This study excluded individuals with a history or current experiences of psychosis, bipolar disorder, personality disorders, or substance abuse within the preceding 6 months. Those with intellectual disability, degenerative neuropsychiatric conditions (such as Huntington disease, Parkinson disease, or dementia), neurological disorders including stroke and epilepsy, severe medical conditions (eg, cancer), or brain injury were also excluded. To maintain sample homogeneity, participants who had previously been exposed to psychiatric medications were also excluded [22].

The HC group was comprised of individuals who did not meet the established diagnostic criteria for MDD or anxiety-related disorders as outlined in the *DSM-*5. These individuals demonstrated normal results in both medical and neurological screening tests.

### Ethical Considerations

This study received approval from the Institutional Review Board of Samsung Medical Center (SMC 2019-07-039-010). This investigation adhered strictly to the registered protocol. No deviations occurred during its implementation. All eligible participants provided written informed consent prior to their inclusion in this study.

Participants were compensated for their time and effort during the study. Specifically, each participant received US $34 for the screening visit and US $68 for each of the intervention visits at weeks 0, 2, and 4. Total compensation amounted to US $238 per participant, ensuring fair and transparent reimbursement for their participation.

All data collected during the study were deidentified prior to analysis to ensure the privacy and confidentiality of participant information. No personally identifiable information was retained or disclosed at any stage of the research.

### Study Designs and Procedures

This study was conducted as a 4-week RCT at the Clinical Study Center of Samsung Medical Center. The efficacy of VR-based BF in the DAS/VR group was compared to that of conventional BF in the DAS/BF group. In addition, the HC/VR group received VR-based BF to evaluate its effects in nonclinical populations. Participants with depressive symptoms, anxiety symptoms, or both were randomly assigned to either DAS/VR group or DAS/BF group at a 1:1 ratio using computer-generated randomized numbers. This study was conducted in an open-label manner; neither the participants nor the individuals delivering the interventions were blinded to group allocation. Randomization was conducted by an independent researcher who was not involved in participant recruitment or outcome assessments, ensuring allocation concealment. To evaluate the efficacy of VR-based BF in healthy subjects, the HC group was recruited with the aim of achieving comparable distributions of age, gender, and education level to those of the DAS/VR group. The HC group received VR-based BF intervention. Including an HC group offers valuable insights into applying the intervention to broader populations, such as those aiming to improve sleep quality for general health instead of clinical treatment. This approach expands the applicability of our findings, offering a better understanding of how VR-based BF may benefit both clinical and nonclinical populations.

To maintain intervention integrity, participants in different intervention groups (DAS/VR and DAS/BF) were scheduled for their sessions at separate times to prevent interaction or exchange of information between groups. This scheduling approach ensured that participants were unaware of the intervention details for other groups, minimizing potential contamination effects. All participants were asked to visit the clinical study center 3 times (at weeks 0, 2, and 4) to receive allocated intervention, followed by interviews to assess the Montgomery-Asberg Depression Rating Scale (MADRS) [23], the State-Trait Anxiety Inventory [24], and the Visual Analog Scale [25] after each session. Although participants visited 3 times, the PSQI was specifically administered in weeks 0 and 4 to evaluate the overall change in sleep quality over the 4-week intervention period. During the VR session, a Samsung Odyssey plus (Samsung Electronics Co., Ltd.) was used with peripheral devices, including a head-mounted display with head tracking and stereo earphones connected. Each participant sat in the motion chair and wore the head-mounted display.

### VR-Based Relaxation Training Protocol

Participants watched and listened to VR relaxation training video comprised of 4 natural scenes for 5 minutes. A psychiatrist (HJJ) led participants through a breathing exercise during the VR session. After an ancient bell rang, participants started to slowly wander through nature in virtual reality while listening to classically soothing background music and digital nature sounds that were specifically designed to go with VR images, such as sounds of birds chirping, wind, water flowing, and rustling leaves specifically designed to accompany VR images. At the same time, guided relaxation therapy was performed (such as, “Relax your muscles. Breathe in slowly while your stomach expands, then exhale one, two, three times. Exhale gradually as your abdomen expands”; Multimedia Appendix 1). While practicing relaxation techniques, participants were permitted to slowly cross a river, soar to skies, and stroll through a tranquil meadow in the VR (Multimedia Appendix 2).

Regarding conventional BF, ProComp Infiniti (Thought Technology, Ltd.), a computerized BF device, was used. Participants were instructed about relaxation techniques while observing physiological parameter signals, which encompassed heart rate/blood pressure, skin conductance, respiration, and skin temperature displayed on the screen with a therapist. During the BF session, the therapist provided feedback to subjects when physiological markers changed by 15%. The BF intervention was carried out for 5 minutes.

### Outcome Measures

Participants were requested to recollect and report their sleep patterns from the preceding month, responding to a set of 19 distinct inquiries related to 7 primary components of sleep, including overall sleep quality, sleep latency, sleep duration, sleep efficiency, sleep disturbance, sleep medicine uses, and day dysfunction due to sleepiness using the PSQI. The PSQI was administered at baseline (wk 0) and postintervention (wk 4).

The PSQI is a robust and widely acknowledged instrument for evaluating self-reported adult sleep quality. It efficaciously gauges 7 primary components; sleep quality, sleep latency, sleep duration, sleep efficiency, sleep disturbance, sleep medicine uses, and day dysfunction, allowing for discernment between “poor” and “good” sleep experiences. Importantly, each of these 7 primary components can be systematically assessed by participants, contributing to a comprehensive and nuanced understanding of an individual’s sleep habits and their implications. These 7 primary components were scored on a Likert scale from 0 to 3, with a score of 0 representing the positive extreme (“not during the past month”) and a score of 3 representing the negative extreme. Each participant received a global PSQI score after adding up scores for the 7 factors (all equally weighted on a scale of 0 to 3). The sum of those scores ranged from 0 to 21, with a higher score indicating a poorer sleep quality. In comparison with clinical and laboratory measurements, a global PSQI score >5 was indicative of poor sleep quality [18].

The test-retest reliability analysis revealed a notable overall correlation coefficient of 0.87 for a PSQI global score among patients afflicted with primary insomnia [26]. Notably, as an indicator of sleep disruptions within the context of patients with insomnia compared to control groups, a PSQI global score exceeding 5 demonstrated a remarkable sensitivity of 98.7% and a specificity of 84.4%. This highlights the practical utility of PSQI in diagnosing sleep disturbances in individuals with insomnia, affirming its significance in clinical and research contexts [26].

### Statistical Analyses

According to a previous study, the sample size was determined based on an effect size of 0.7 in MADRS with a type I error rate of 0.05 and a power of 80%. Accounting for a dropout rate of 15%, we planned to recruit 120 participants [27]. This study used the same cohort as Cho et al (2024) and used PSQI as the primary outcome. Although the sample size calculation was originally based on MADRS, the current sample size was considered sufficient for detecting meaningful differences in PSQI with a power of 79.75% (effect size=0.1) from post-hoc power analysis.

To analyze distribution of clinical and demographic characteristics of each group (DAS/VR, DAS/BF, HC/VR), descriptive statistics are presented as mean with SD for continuous variables and as frequency with percentage for categorical variables. Continuous variables included age, education (years), Barratt Impulsiveness Scale-11 [28], and Global PSQI score. Categorical variables consisted of sex (male, female), drinking frequency (no, yes), smoking status (never, current), and Clinical Global Impression-Severity categories (not ill, minimally ill, mildly ill). Group comparisons among the 3 groups were conducted using the *χ*^2^ test or Fisher exact test for categorical variables. For continuous variables, they were compared using one-way ANOVA or Kruskal–Wallis test according to satisfaction of normality assumption by Shapiro–Wilks test and equality of variance by Levene test. Changes in outcome measurements between baseline (0 wk) and 4-week follow-up visit were examined via paired *t* test or Wilcoxon signed-rank test in each group. For changes in outcomes, pairwise comparisons were focused on DA/VR versus DA/BF and DAS/VR versus HC/VR. They were conducted using either Wilcoxon rank-sum test or *t* test, depending on data distribution. After adjusting for sex and age, we additionally performed linear regression analysis to compare between groups. Linear regression analysis was conducted to assess changes in global PSQI scores between groups (DAS/VR, DAS/BF, and HC/VR). The dependent variable was the change in global PSQI score from baseline to week 4. Independent variables included group, age, and sex, selected based on theoretical relevance to sleep quality outcomes. No stepwise variable selection (forward or backward) was applied. Statistical significance was considered when *P* value was less than .05. All statistical analyses were performed with SAS version 9.4 (SAS Institute).

## Results

### Baseline Characteristics of Participants

**Figure 1.**
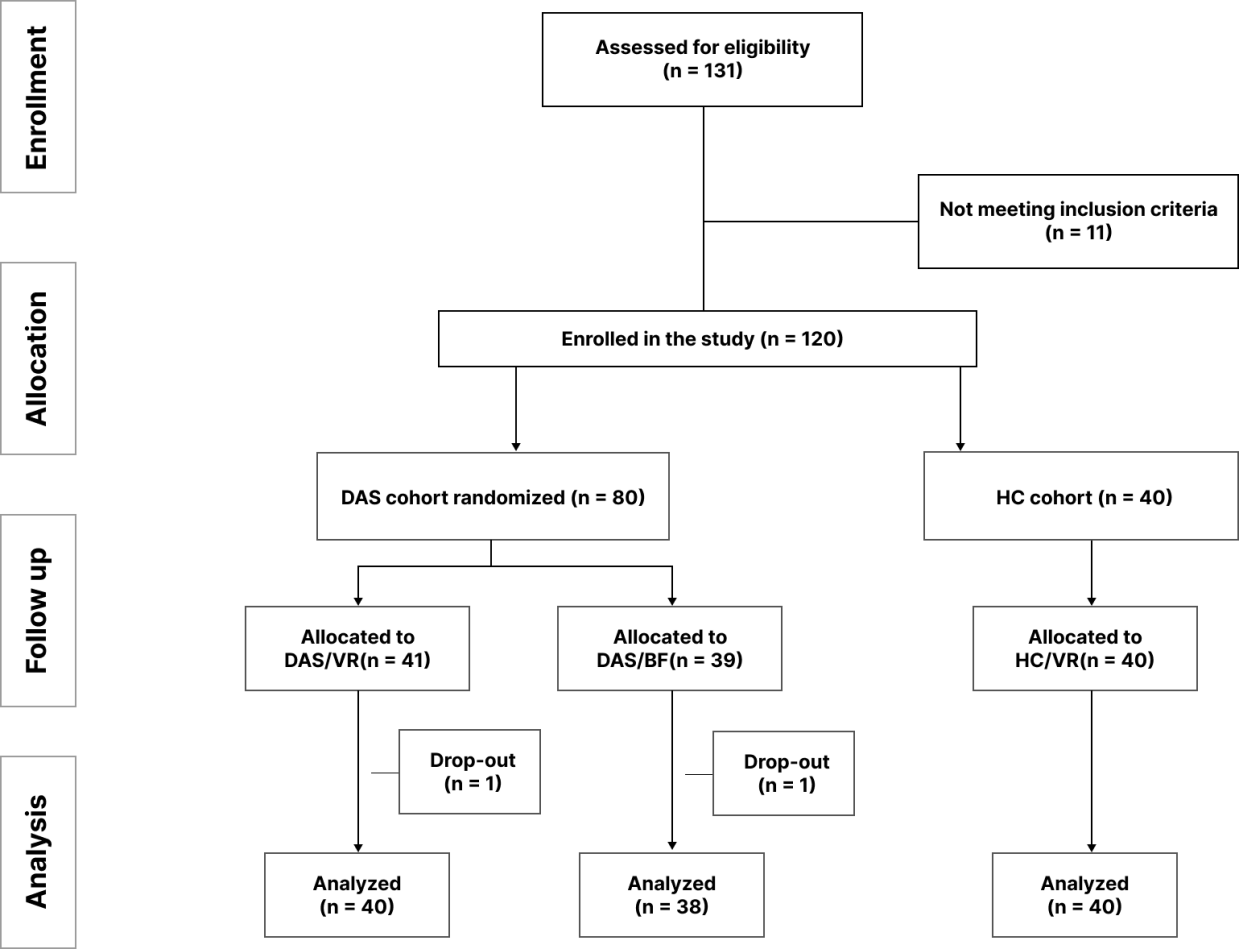
CONSORT (Consolidated Standards of Reporting Trials) flow diagram illustrating the recruitment, allocation, follow-up, and analysis of study participants. A total of 131 individuals were assessed for eligibility. Of them, 11 were excluded for not meeting inclusion criteria. Of 120 participants enrolled, 80 were randomized into a cohort with DAS. They were further allocated into DAS/VR (n=41) and DAS/BF (n=39) groups. The remaining 40 participants were assigned to the HC cohort with VR-based BF (HC/VR). Dropouts occurred in both DAS/VR (n=1) and DAS/BF (n=1) groups. BF: biofeedback; DAS: depressive symptoms, anxiety symptoms, or both; HC: healthy control; VR: virtual reality.

Between December 2019 and February 2022, a total of 131 self-referred adults aged 18 years and older were enrolled in this study through advertising at Samsung Medical Center in Seoul, South Korea. The screening process resulted in exclusion of 13 volunteers due to psychiatric diagnoses, including 9 with MDDs, 3 with comorbid phobic disorders, and 1 with antisocial personality disorder. A total of 120 participants were enrolled for this study, including 80 participants in the DAS group and 40 participants in the HC group. Those in the DAS group were allocated into a VR intervention group (n=41) and a BF intervention group (n=39) at random. After the first visit, one person in the VR group and one person in the BF group withdrew from this study. Finally, a total of 118 participants were subjected to comprehensive analysis (Figure 1). Demographic details and initial clinical measurements of these participants are shown in Table 1. All participants were Korean. Age distribution across groups was as follows. The DAS/VR group had a mean age of 45.1 (SD 10.2) years. The DAS/BF group had a mean age of 42.9 (SD 10.6) years and the HC/VR group had a mean age of 40.3 (SD 12) years. Gender distribution included 20% (8/40) males and 80% (32/40) females in the DAS/VR group, 21.1% (8/38) males and 79% (30/38) females in the DAS/BF group, and 40% (16/40) males and 60% (24/40) females in the HC/VR group. Differences in age, sex, education, drinking frequency, and smoking were not significant between groups. However, the mean global PSQI score of participants at baseline was 9.7 (SD 2.5) for the DAS/VR group, 10.8 (SD 2.8) for the DAS/BF group, and 5.9 (SD 2.4) for the HC/VR group, showing a significant difference (*P*<.001).

**Table 1.** Baseline characteristics of participants.

	Depressive symptoms, anxiety symptoms, or both (DAS)		Healthy control (HC)	*P* value
	DAS/VR^a^ group (n=40)	DAS/BF^b^ group (n=38)	HC/VR group (n=40)	
Age (years), mean (SD)	45.1 (10.2)	42.9 (10.6)	40.3 (12)	.15
Sex, n (%)				.08
Male	8 (20)	8 (21.1)	16 (40)	
Female	32 (80)	30 (79)	24 (60)	
Education (years), mean (SD)	14.8 (2.1)	14.8 (1.8)	15.3 (2.1)	.31
Drinking frequency, n (%)				.49
No	11 (27.5)	15 (39.5)	12 (30)	
Yes	29 (72.5)	23 (60.5)	28 (70)	
Smoking, n (%)				.16
Never	36 (90)	28 (73.7)	30 (76.9)	
Current	4 (10)	10 (26.3)	9 (23.1)	
BIS-11^c^, mean (SD)	63.2 (10.1)	68.9 (7.9)	57.4 (7.8)	<.001
CGI-S^d^, n (%)				<.001^e^
Not ill	23 (57.5)	18 (47.4)	37 (92.5)	
Minimally ill	15 (37.5)	16 (42.1)	3 (7.5)	
Mildly ill	2 (5)	4 (10.5)	0 (0)	
Global PSQI^f^ score at baseline, mean (SD)	9.7 (2.5)	10.8 (2.8)	5.9 (2.4)	<.001^g^

a VR: virtual reality.

b BF: biofeedback.

c BIS-11: Barratt Impulsiveness Scale-11.

d CGI-S: Clinical Global Impression-Severity.

e *P* value was determined by Fisher exact test; others by Chi-square test for categorical variables.

f PSQI: Pittsburgh Sleep Quality Index.

g *P* value was determined by Kruskal-Wallis test; others by one-way ANOVA for continuous variables.

### Significance of Change in PSQI in Each Group (0 Weeks and 4 Weeks)

**Figure 2.**
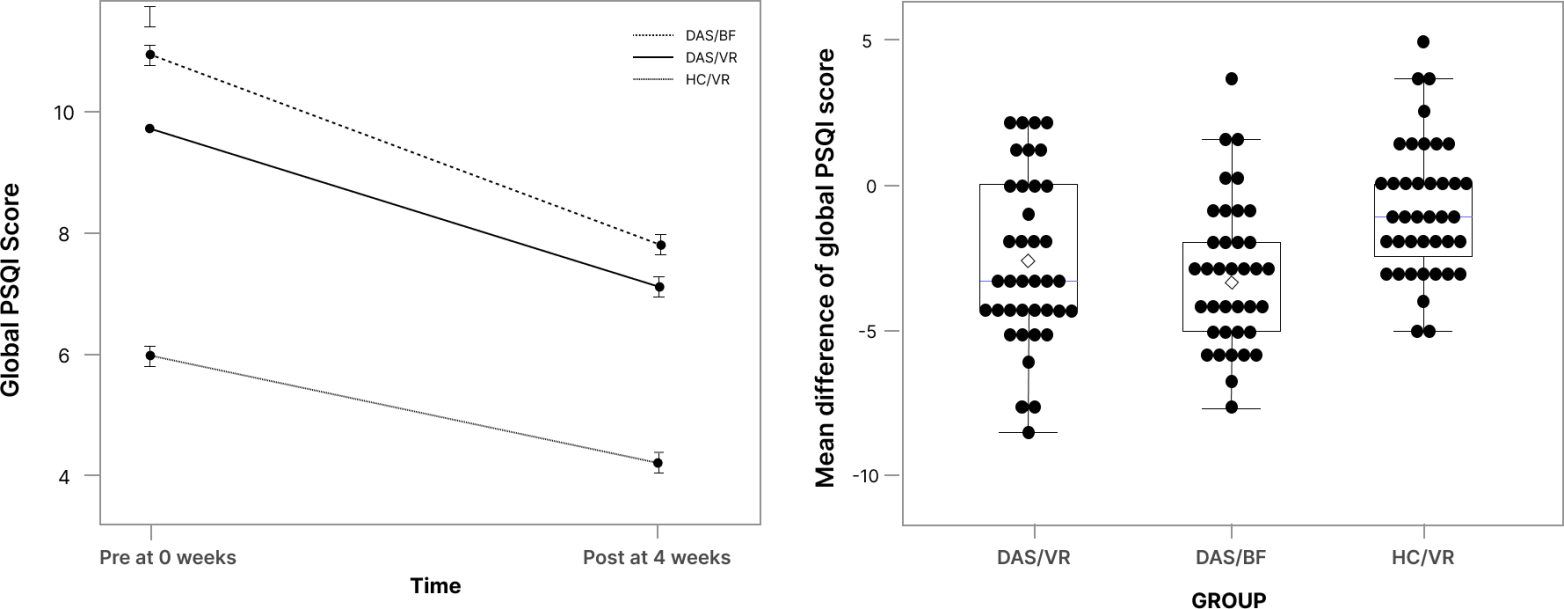
Changes in global PSQI scores over 4 weeks for the 3 groups (DAS/VR, DAS/BF, and HC/VR). BF: biofeedback; DAS: depressive symptoms, anxiety symptoms, or both; HC: healthy control; PSQI: Pittsburgh Sleep Quality Index; VR: virtual reality.

Pre- and postintervention mean (SD) PSQI scores at 0 week and 4 weeks for the 3 groups (DAS/VR, DAS/BF, and HC/VR) are presented in Table 2. Pre- and postintervention Global PSQI score showed significant reductions across all 3 groups. In the DAS/VR group, the mean Global PSQI score decreased significantly from baseline (9.70, SD 2.49) to 4 weeks (7.20, SD 2.46; *P*<.001). Similarly, the DAS/BF group exhibited a significant (*P*<.001) reduction in the Global PSQI score from 10.76 (SD 2.76) to 7.37 (SD 2.28). The HC/VR group also showed a significant decrease, with the Global PSQI score significantly (*P*=.01) improving from 5.85 (SD 2.39) to 4.90 (SD 2.11) (Figure 2).

**Figure 3.**
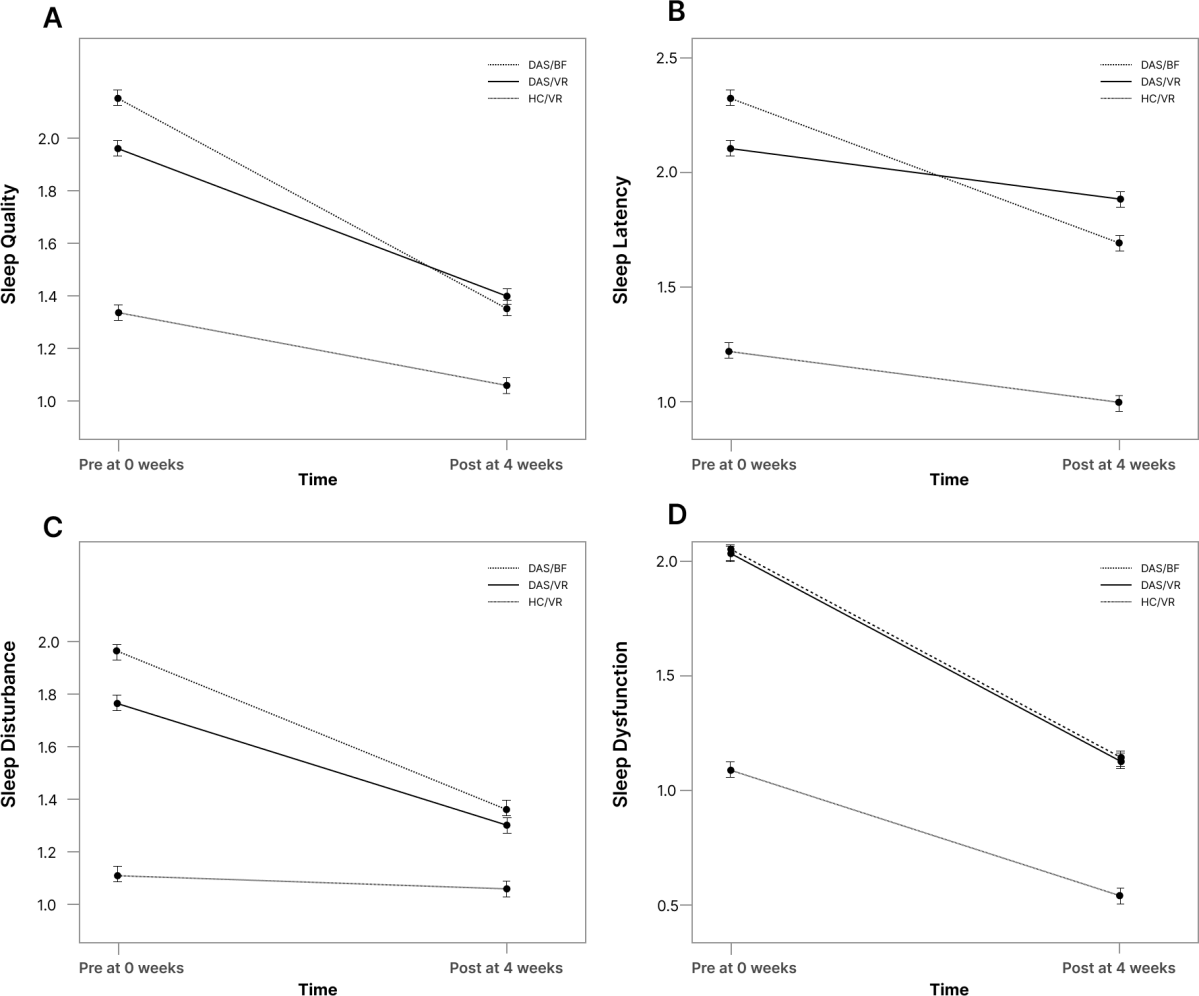
Significant improvements in sleep quality measures across the 3 groups (DAS/VR, DAS/BF, and HC/VR) in 4 PSQI components: (A) sleep quality, (B) sleep latency, (C) sleep disturbance, and (D) daytime dysfunction. BF: biofeedback; DAS: depressive symptoms, anxiety symptoms, or both; HC: healthy control; PSQI: Pittsburgh Sleep Quality Index; VR: virtual reality.

Regarding daytime dysfunction, all groups demonstrated significant (*P*<.001) improvements from baseline to 4 weeks. Similar trends were observed for other PSQI components such as sleep quality, sleep latency, and sleep disturbance, all showing marked improvements in both DAS/VR and DAS/BF groups, whereas changes of the HC/VR group did not reach statistical significance (Figure 3). These findings suggest that the intervention is efficacious in enhancing various aspects of sleep quality, particularly for DAS groups.

The PSQI score at preintervention (0 wk) and postintervention (4 wk) are presented as mean (SD). *P* values are determined by paired *t* test or Wilcoxon signed-rank test according to normality assumption.

**Table 2.** Significance of change in PSQI^a^ score in each group (0 wk and 4 wk). The PSQI score at preintervention (0 wk) and postintervention (4 wk) are presented as mean (SD).

	Depressive symptoms, anxiety symptoms, or both (DAS)						Healthy control (HC)		
	DAS/VR^b^ group (n=40)			DAS/BF^c^ group (n=38)			HC/VR^b^ group (n=40)		
	0 weeks, mean (SD)	4 weeks, mean (SD)	*P* value	0 weeks, mean (SD)	4 weeks, mean (SD)	*P* value	0 weeks, mean (SD)	4 weeks, mean (SD)	*P* value
PSQI component scores									
Sleep quality	1.90 (0.44)	1.50 (0.64)	=0.0010	2.08 (0.36)	1.45 (0.60)	<.001	1.30 (0.65)	1.13 (0.52)	.19
Sleep latency	2.10 (0.71)	1.60 (0.74)	<.001	2.24 (0.75)	1.47 (0.80)	<.001	1.25 (0.84)	1.0 (0.82)	.06
Sleep duration	1.40 (0.84)	1.25 (0.87)	.30	1.66 (0.81)	1.42 (0.86)	.18	0.88 (0.72)	0.98 (0.70)	.42
Sleep efficiency	0.40 (0.74)	0.43 (0.87)	>.99	0.79 (1.04)	0.55 (0.8)	.25	0.23 (0.66)	0.20 (0.46)	.95
Sleep disturbance	1.78 (0.62)	1.20 (0.46)	<.001	1.89 (0.61)	1.24 (0.54)	<.001	1.05 (0.50)	0.98 (0.42)	.55
Sleep medicine uses	0.05 (0.22)	0.00 (0.00)	.50	0.03 (0.16)	0.00 (0.00)	>.99	0.00 (0.00)	0.00 (0.00)	NA^d^
Daytime dysfunction	2.08 (0.73)	1.23 (0.86)	<.001	2.08 (0.78)	1.24 (0.71)	<.001	1.15 (0.80)	0.63 (0.74)	<.001
Global PSQI score	9.70 (2.49)	7.20 (2.46)	<.001	10.76 (2.76)	7.37 (2.28)	<.001^e^	5.85 (2.39)	4.90 (2.11)	.01^e^

a PSQI: Pittsburgh Sleep Quality Index.

b VR: virtual reality.

c BF: biofeedback.

d Not available.

e *P* values are determined by paired *t* test; others by Wilcoxon signed-rank test.

### Pairwise Comparison of Changes in PSQI Score Between Pre- and Postintervention

Table 3 presents mean changes at the last session (postintervention at wk 4) compared to baseline (preintervention at wk 0) analyzed by Wilcoxon rank-sum test or *t* test for 2 pairwise comparisons between VR and BF in DAS and between DAS/VR and HC/VR in each group. Further analysis was also performed for adjusted pairwise comparisons of the PSQI score between different groups. According to linear regression analysis that was adjusted for age and sex, there was no significant difference in improvement of the PSQI score following VR or BF intervention between DAS/VR and DAS/BF groups. In contrast, there were significant differences in components of sleep disturbance and global PSQI score between DAS/VR and HC/VR groups (all *P*<.05).

Both DAS/VR and DAS/BF groups demonstrated notable improvements in sleep disturbance, showing reductions of −0.58 (SD 0.75) and −0.66 (SD 0.75), respectively. However, the difference in the reduction of sleep disturbance between the 2 groups was not statistically significant (*P*=.49). When comparing the DAS/VR group to the HC/VR group, the DAS/VR group exhibited a marked and statistically significant improvement in sleep disturbance (*P*<.01). The global PSQI score showed notable improvements in both DAS/VR and DAS/BF groups, with decreases of −2.5 (SD 2.89) and −3.39 (SD 2.80), respectively. Nevertheless, the global PSQI score decrease did not differ statistically significantly between DAS/VR and DAS/BF groups (*P*=.14), although DAS/VR and HC/VR groups showed statistically significant (*P*=.01) difference in global PSQI score.

**Table 3.** Pairwise comparison of changes in Pittsburgh Sleep Quality Index (PSQI) score between pre- and postintervention. The change was calculated as the difference between postintervention and preintervention and presented as mean (SD).

	The change in the PSQI score, mean (SD)			Pairwise comparison, *P* value^a^		Adjusted pairwise comparison, *P* value^b^	
	DAS^c^/VR^d^ group (n=40)	DAS/BF^e^ group (n=38)	HC^f^/VR group (n=40)	DAS/VR vs DAS/BF	DAS/VR vs HC/VR	DAS/VR vs DAS/BF	DAS/VR vs HC/VR
PSQI component scores							
Sleep quality	−0.4 (0.67)	−0.63 (0.67)	−0.18 (0.71)	.15	.18	.15	.21
Sleep latency	−0.5 (0.72)	−0.76 (0.79)	−0.25 (0.74)	.10	.14	.13	.14
Sleep duration	−0.15 (0.80)	−0.24 (0.94)	0.1 (0.59)	.54	.13	.57	.10
Sleep efficiency	0.03 (1.05)	−0.24 (1.10)	−0.03 (0.80)	.46	.67	.24	.81
Sleep disturbance	−0.58 (0.75)	−0.66 (0.75)	−0.08 (0.53)	.78	=0.0007	.49	=0.0014
Sleep medicine uses	−0.05 (0.22)	−0.03 (0.16)	0.00 (0.00)	.60	.16	.58	.10
Daytime dysfunction	−0.85 (1.19)	−0.84 (0.68)	−0.53 (0.78)	.84	.14	.97	.24
Global PSQI score	−2.50 (2.89)	−3.39 (2.80)	−0.95 (2.09)	.20	.01	.14	.01

a *P* values in univariable analyses were determined by Wilcoxon rank-sum test according to the normality assumption.

b *P* values in multivariable analyses were determined by linear regression analysis after adjusting for age and sex.

c DAS: Group with depressive symptoms, anxiety symptoms, or both.

d VR: virtual reality.

e BF: biofeedback.

f HC: healthy control.

### Participant-Reported Adverse Events

While VR-based BF was generally well tolerated, some participants reported minor adverse events, including visual discomfort, difficulty adjusting the VR headset, mild dizziness, and occasional neck strain due to weight of the equipment. A small number of participants experienced anxiety triggered by specific VR scenarios, such as height-related scenes. Although these events did not significantly impact the study’s outcomes, they highlight the need for ergonomic improvements to the VR headset and enhanced content design to minimize motion sickness and anxiety triggers. Addressing these issues in future iterations could improve participants’ comfort and engagement.

## Discussion

### Principal Findings

This RCT assessed the efficacy of VR-based BF in comparison with conventional BF in improving sleep quality of individuals with depressive symptoms, anxiety symptoms, or both according to pre- and postintervention mean scores of PSQI. Our results demonstrated significant improvements in sleep quality, latency, disturbance, daytime dysfunction, and global PSQI scores in DAS/VR and DAS/BF groups. VR enables users to create interactive environments that can influence physiological and emotional responses [15]. In addition to sleep quality improvements, our primary outcome analysis revealed consistent decreases in both clinician-rated depression scores and self-reported symptoms of depression and anxiety across all visits (at weeks 0, 2, and 4) [27].

The relationship between sleep disorders and depression has been well documented [29], with a clear association established between poor sleep status and psychological issues [30]. Sleep quality is inversely linked to depression, physical symptoms, and anxiety, highlighting its importance for mental health and daily functioning [31]. These findings suggest that improvements in sleep quality observed in this study are accompanied by significant alleviation of depressive symptoms, anxiety symptoms, or both, underscoring that VR-based BF potentially has dual benefits as a therapeutic intervention. Such dual benefits highlight the promise of VR-based BF as a tool not only for enhancing sleep quality, but also for addressing underlying psychological symptoms. It is particularly valuable for clinical populations with comorbid depressive symptoms, anxiety symptoms, or both.

### Comparative Efficacy of VR-Based BF on Sleep Quality

Improvements in sleep quality observed in our study align with previous research demonstrating the benefits of BF interventions for insomnia, as BF has been shown to alleviate insomnia symptoms, reduce anxiety and depression, and enhance subjective sleep quality [9]. A 6-week BF protocol can significantly improve insomnia, sleep quality, duration, and daytime dysfunction in individuals with comorbid MDD and insomnia based on psychological and physiological assessments [9]. A pilot RCT has found that neurofeedback and cognitive behavioral therapy for insomnia are equally efficacious for insomnia [32], while other studies have highlighted the potential of BF for treating comorbid insomnia and related disorders [9,33]. Similarly, a mobile BF intervention has been shown to efficaciously reduce anxiety and improve sleep quality in healthy adults [34]. One study has demonstrated the efficacy of VR natural environments in the context of stress reduction and relaxation, underscoring their viability as therapeutic tools [35]. Our study builds on these findings by incorporating VR technology, which can enhance engagement and potentially improve adherence to intervention.

Compared to prior research focusing solely on BF [9,36-39], our study demonstrated that VR-based BF not only improved sleep quality in individuals with depressive symptoms, anxiety symptoms, or both, but also had a measurable impact on nonclinical populations, such as those in the HC/VR group. This finding is consistent with earlier studies that have highlighted the potential of VR-based relaxation tools to improve stress and sleep outcomes in healthy individuals [11,12,40]. However, improvements in the HC/VR group were less pronounced than those in DAS groups, suggesting that VR-based BF might be particularly beneficial for clinical populations.

This study advances the field by being one of the few RCTs evaluating the efficacy of VR-based BF for sleep quality, specifically in individuals with depressive symptoms, anxiety symptoms, or both. In contrast to a previous study that primarily addressed depression and anxiety [27], our study focused on the often-overlooked aspect of sleep quality in mental health interventions. This investigation demonstrated the potential of VR technology to enhance therapeutic interventions, particularly for individuals exhibiting reduced engagement with conventional BF approaches.

### Limitations

This study has several limitations that should be addressed in future research. First, the single-center design might have limited the generalizability of our findings. Expanding this study to include multiple centers and diverse populations would provide a broader perspective on the efficacy of VR-based BF. Second, participants of this study were self-referred. They responded voluntarily to recruitment advertisements. This recruitment method might have introduced selection bias since self-referred individuals might differ from clinically referred patients in important ways, such as symptom severity, treatment history, or motivation to participate in interventions. Consequently, findings of this study might not be fully generalized to broader clinical populations. Future studies should consider recruiting participants from diverse sources, including direct clinical referrals, to ensure a more representative sample and enhance external validity of results. Due to complexities of managing depression, careful monitoring of patients undergoing treatment is crucial to ensure efficacious and sustained improvements. Third, the classification of participants into the DAS group was based solely on self-reported screening instruments (PHQ-9 and PDSS) rather than structured clinical interviews, which are considered the gold standard for psychiatric diagnosis. This reliance on self-report measures may limit the diagnostic accuracy and generalizability of the findings. Fourth, sleep quality was evaluated exclusively through the subjective PSQI measure without the use of objective assessments such as polysomnography or actigraphy. The lack of objective sleep evaluations may introduce measurement bias and limit the ability to fully capture physiological changes related to sleep quality. Future research should incorporate structured clinical interviews and objective sleep measures to enhance diagnostic validity and methodological rigor.

Despite these limitations, our findings have significant implications for clinical practice. VR-based BF offers a flexible and accessible alternative to conventional BF, allowing patients to engage in therapeutic sessions remotely under medical supervision [41]. This approach can reduce the reliance on highly trained professionals while maintaining therapeutic efficacy, making it a practical option for diverse health care settings. Moreover, minimal apparent side effects associated with VR-based BF position it as a safe and innovative intervention for addressing sleep disturbances, particularly for individuals with depressive and anxiety symptoms [11,12,41].

### Conclusions

In conclusion, this study highlights the efficacy of both VR-based BF and conventional BF with a therapist as therapeutic approaches for improving sleep quality in individuals with DAS. While our results showed no significant differences between the 2 intervention methods, both demonstrated meaningful improvements in sleep quality parameters compared to baseline. By integrating VR technology, the therapeutic experience can be enhanced, offering an alternative to conventional methods that may be particularly engaging for some patients. Future research should aim to validate these findings across diverse populations and explore long-term effects to further establish the role of these interventions in clinical practice.

## Supplementary material

10.2196/65772 Multimedia Appendix 1 Structured overview of the virtual reality–based relaxation intervention protocol.

10.2196/65772 Multimedia Appendix 2 Visual representations of the virtual reality–based relaxation intervention protocol.

10.2196/65772 Checklist 1 CONSORT-EHEALTH (V 161) checklist. CONSORT: Consolidated Standards of Reporting Trials.

## Abbreviations

BF: biofeedback
DAS: depressive symptoms, anxiety symptoms, or both
DSM-5: Diagnostic and Statistical Manual of Mental Disorders, Fifth Edition
HC: healthy control
MADRS: Montgomery-Asberg Depression Rating Scale
MDD: major depressive disorder
PDSS: Panic Disorder Severity Scale
PHQ-9: Patient Health Questionnaire-9
PSQI: Pittsburgh Sleep Quality Index
RCT: randomized controlled trial
VR: virtual reality
