# Supplementary material for: Impact of Virtual Reality–Based Biofeedback on Sleep Quality Among Individuals With Depressive Symptoms, Anxiety Symptoms, or Both: 4-Week Randomized Controlled Study
Source: J Med Internet Res. 2025 Jun 20;27:e65772. doi: 10.2196/65772 (PMC12204043; doi:10.2196/65772)
Supplement: Multimedia Appendix 1 [file jmir-v27-e65772-s001.docx]

**Multimedia Appendix 1.** Structured Overview of the VR-Based Relaxation Intervention Protocol.

| **Time** | **Activity** | **Instructor** | **Details** |
| --- | --- | --- | --- |
| 0 – 45 s | Introduction to VR session | Psychiatrist (HJJ) | Participants are seated, wearing the head-mounted display (HMD), and are briefed about the session |
| 46 s –  2 min 24 s | Breathing exercise | Psychiatrist (HJJ) | Guided breathing exercise (e.g., "Breathe in slowly while your stomach expands, then exhale slowly.") |
| 2 min 25 s – 4 min 21 s | Transition to VR relaxation experience | Psychiatrist (HJJ) | Participants hear an ancient bell sound, signaling the start of the VR experience |
| 4 min 22 s – 7 min 59 s | Immersion in VR nature scenes | Psychiatrist (HJJ) | Participants wander through four nature scenes (forest, valley, ocean, meadow) with background music and nature sounds (birds chirping, wind, and flowing water) |
| 8 min –  8 min 58 s | Guided relaxation therapy | Psychiatrist (HJJ) | Relaxation instructions are synchronized with the VR experience (e.g., "Relax your muscles, cross a river, stroll through a meadow") |
