# Supplementary material for: Impact of Virtual Reality–Based Biofeedback on Sleep Quality Among Individuals With Depressive Symptoms, Anxiety Symptoms, or Both: 4-Week Randomized Controlled Study
Source: J Med Internet Res. 2025 Jun 20;27:e65772. doi: 10.2196/65772 (PMC12204043; doi:10.2196/65772)
Supplement: Multimedia Appendix 2 [file jmir-v27-e65772-s002.docx]

**Multimedia Appendix 2.** Visual Representations of the VR-Based Relaxation Intervention Protocol.


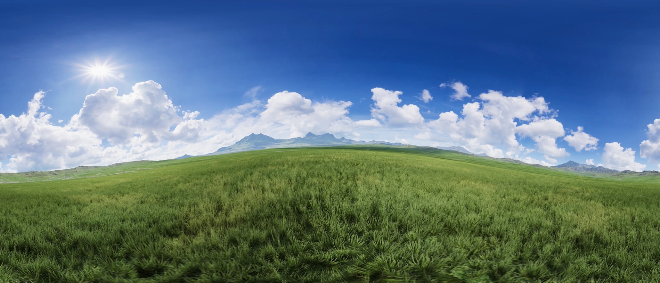

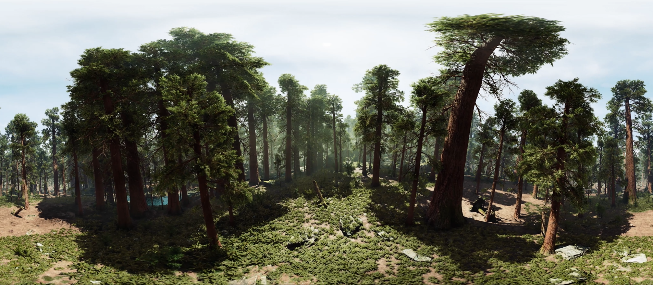


(A) (B)


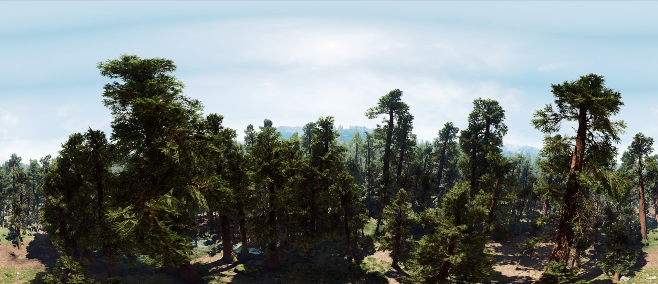

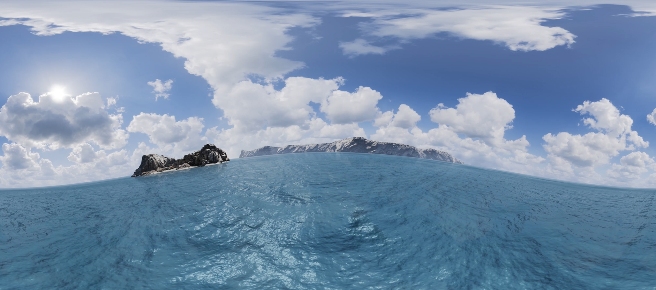


(C) (D)

(A) Introduction to VR session; Participants are seated, wear the head-mounted display (HMD), and are introduced to the VR environment. (B) Breathing exercise: Guided breathing activity led by a psychiatrist to prepare participants for relaxation. (C) Transition to VR relaxation experience: Participants explore a serene virtual forest with ambient sounds designed for relaxation. (D) Immersion in VR nature scenes: A calming VR experience of the ocean, with flowing water sounds to enhance relaxation.
