# Supplementary material for: Impact of Virtual Reality–Based Biofeedback on Sleep Quality Among Individuals With Depressive Symptoms, Anxiety Symptoms, or Both: 4-Week Randomized Controlled Study
Source: J Med Internet Res. 2025 Jun 20;27:e65772. doi: 10.2196/65772 (PMC12204043; doi:10.2196/65772)
Supplement: Checklist 1 [file jmir-v27-e65772-s003.pdf]

CONSORT-EHEALTH (V 1.6.1) - Submission/Publication Form

The CONSORT-EHEALTH checklist is intended for authors of randomized trials evaluating web-based and Internet-based applications/interventions, including mobile interventions, electronic games (incl multiplayer games), social media, certain telehealth applications, and other interactive and/or networked electronic applications. Some of the items (e.g. all subitems under item 5 - description of the intervention) may also be applicable for other study designs.

The goal of the CONSORT EHEALTH checklist and guideline is to be
a) a guide for reporting for authors of RCTs,
b) to form a basis for appraisal of an ehealth trial (in terms of validity)

CONSORT-EHEALTH items/subitems are MANDATORY reporting items for studies published in the Journal of Medical Internet Research and other journals / scientific societies endorsing the checklist.

Items numbered 1., 2., 3., 4a., 4b etc are original CONSORT or CONSORT-NPT (non-pharmacologic treatment) items.
Items with Roman numerals (i., ii, iii, iv etc.) are CONSORT-EHEALTH extensions/clarifications.

As the CONSORT-EHEALTH checklist is still considered in a formative stage, we would ask that you also RATE ON A SCALE OF 1-5 how important/useful you feel each item is FOR THE PURPOSE OF THE CHECKLIST and reporting guideline (optional).

Mandatory reporting items are marked with a red *.
In the textboxes, either copy & paste the relevant sections from your manuscript into this form - please include any quotes from your manuscript in QUOTATION MARKS,
or answer directly by providing additional information not in the manuscript, or elaborating on why the item was not relevant for this study.

YOUR ANSWERS WILL BE PUBLISHED AS A SUPPLEMENTARY FILE TO YOUR PUBLICATION IN JMIR AND ARE CONSIDERED PART OF YOUR PUBLICATION (IF ACCEPTED).
Please fill in these questions diligently. Information will not be copyedited, so please use proper spelling and grammar, use correct capitalization, and avoid abbreviations.

DO NOT FORGET TO SAVE AS PDF _AND_ CLICK THE SUBMIT BUTTON SO YOUR ANSWERS ARE IN OUR DATABASE !!!

Citation Suggestion (if you append the pdf as Appendix we suggest to cite this paper in the caption):
Eysenbach G, CONSORT-EHEALTH Group
CONSORT-EHEALTH: Improving and Standardizing Evaluation Reports of Web-based and Mobile Health Interventions
J Med Internet Res 2011;13(4):e126

URL: http://www.jmir.org/2011/4/e126/
doi: 10.2196/jmir.1923
PMID: 22209829

sisu.seong@gmail.com 계정 전환

비공개

초안 저장됨

* 표시는 필수 질문임

Your name *

First Last

Sisu Seong

Primary Affiliation (short), City, Country *

University of Toronto, Toronto, Canada

Sungkyunkwan University, Seoul, South Korea

Your e-mail address *

abc@gmail.com

sisuks@g.skku.edu

Title of your manuscript *

Provide the (draft) title of your manuscript.

Impact of virtual reality-based biofeedback on sleep quality among individuals with depressive symptoms, anxiety symptoms, or both: A 4-week randomized controlled study

Name of your App/Software/Intervention *

If there is a short and a long/alternate name, write the short name first and add the long name in brackets.

VR-based BF(Virtual reality-based biofeedback

Evaluated Version (if any)

e.g. "V1", "Release 2017-03-01", "Version 2.0.27913"

내 답변

Language(s) *

What language is the intervention/app in? If multiple languages are available, separate by comma (e.g. "English, French")

Korean

URL of your Intervention Website or App

e.g. a direct link to the mobile app on app in appstore (itunes, Google Play), or URL of the website. If the intervention is a DVD or hardware, you can also link to an Amazon page.

내 답변

URL of an image/screenshot (optional)

내 답변

Accessibility *

Can an enduser access the intervention presently?

- ○ access is free and open
- ◉ access only for special usergroups, not open
- ○ access is open to everyone, but requires payment/subscription/in-app purchases
- ○ app/intervention no longer accessible
- ○ 기타:

Primary Medical Indication/Disease/Condition *

e.g. "Stress", "Diabetes", or define the target group in brackets after the condition, e.g. "Autism (Parents of children with)", "Alzheimers (Informal Caregivers of)"

Depression (Insomnia)

Primary Outcomes measured in trial *

comma-separated list of primary outcomes reported in the trial

Efficacy of VR-based BF on PSQI, Sleep quality

Secondary/other outcomes

Are there any other outcomes the intervention is expected to affect?

내 답변

Recommended "Dose" *

What do the instructions for users say on how often the app should be used?

- ○ Approximately Daily
- ○ Approximately Weekly
- ○ Approximately Monthly
- ○ Approximately Yearly
- ○ "as needed"
- ◉ 기타: three times (at weeks 0, 2, and 4) to receive the allocated intervention

Approx. Percentage of Users (starters) still using the app as recommended after 3 months *

- ◉ unknown / not evaluated
- ○ 0-10%
- ○ 11-20%
- ○ 21-30%
- ○ 31-40%
- ○ 41-50%
- ○ 51-60%
- ○ 61-70%
- ○ 71%-80%
- ○ 81-90%
- ○ 91-100%
- ○ 기타:

Overall, was the app/intervention effective? *

- ( ) yes: all primary outcomes were significantly better in intervention group vs control
- (•) partly: SOME primary outcomes were significantly better in intervention group vs control
- ( ) no statistically significant difference between control and intervention
- ( ) potentially harmful: control was significantly better than intervention in one or more outcomes
- ( ) inconclusive: more research is needed
- ( ) 기타:

Article Preparation Status/Stage *

At which stage in your article preparation are you currently (at the time you fill in this form)

- ( ) not submitted yet - in early draft status
- ( ) not submitted yet - in late draft status, just before submission
- ( ) submitted to a journal but not reviewed yet
- ( ) submitted to a journal and after receiving initial reviewer comments
- (•) submitted to a journal and accepted, but not published yet
- ( ) published
- ( ) 기타:

Journal *

If you already know where you will submit this paper (or if it is already submitted), please provide the journal name (if it is not JMIR, provide the journal name under "other")

- ○ not submitted yet / unclear where I will submit this
- ◉ Journal of Medical Internet Research (JMIR)
- ○ JMIR mHealth and UHealth
- ○ JMIR Serious Games
- ○ JMIR Mental Health
- ○ JMIR Public Health
- ○ JMIR Formative Research
- ○ Other JMIR sister journal
- ○ 기타:

Is this a full powered effectiveness trial or a pilot/feasibility trial? *

- ○ Pilot/feasibility
- ◉ Fully powered

Manuscript tracking number *

If this is a JMIR submission, please provide the manuscript tracking number under "other" (The ms tracking number can be found in the submission acknowledgement email, or when you login as author in JMIR. If the paper is already published in JMIR, then the ms tracking number is the four-digit number at the end of the DOI, to be found at the bottom of each published article in JMIR)

- ○ no ms number (yet) / not (yet) submitted to / published in JMIR
- ◉ 기타: 65772

TITLE AND ABSTRACT

1a) TITLE: Identification as a randomized trial in the title

1a) Does your paper address CONSORT item 1a? *

I.e does the title contain the phrase "Randomized Controlled Trial"? (if not, explain the reason under "other")

- (x) yes
- ( ) 기타:

1a-i) Identify the mode of delivery in the title

Identify the mode of delivery. Preferably use “web-based” and/or “mobile” and/or “electronic game” in the title. Avoid ambiguous terms like “online”, “virtual”, “interactive”. Use “Internet-based” only if Intervention includes non-web-based Internet components (e.g. email), use “computer-based” or “electronic” only if offline products are used. Use “virtual” only in the context of “virtual reality” (3-D worlds). Use “online” only in the context of “online support groups”. Complement or substitute product names with broader terms for the class of products (such as “mobile” or “smart phone” instead of “iphone”), especially if the application runs on different platforms.

	1	2	3	4	5	
subitem not at all important	( )	( )	( )	( )	(x)	essential

선택해제

Does your paper address subitem 1a-i? *

Copy and paste relevant sections from manuscript title (include quotes in quotation marks "like this" to indicate direct quotes from your manuscript), or elaborate on this item by providing additional information not in the ms, or briefly explain why the item is not applicable/relevant for your study

Impact of virtual reality-based biofeedback on sleep quality among individuals with depressive symptoms, anxiety symptoms, or both: A 4-week randomized controlled study

1a-ii) Non-web-based components or important co-interventions in title

Mention non-web-based components or important co-interventions in title, if any (e.g., “with telephone support”).

	1	2	3	4	5	
subitem not at all important	○	○	○	◉	○	essential

선택해제

Does your paper address subitem 1a-ii?

Copy and paste relevant sections from manuscript title (include quotes in quotation marks "like this" to indicate direct quotes from your manuscript), or elaborate on this item by providing additional information not in the ms, or briefly explain why the item is not applicable/relevant for your study

Yes, the paper partially addresses subitem 1a-ii. The manuscript title, "Efficacy of Virtual Reality-Based Biofeedback on Sleep Quality in Individuals with Depression or Anxiety Symptoms: A Randomized Controlled Trial", specifies the main intervention as Virtual Reality-Based Biofeedback. However, the title does not include explicit details about non-web-based components or any additional co-interventions, such as the involvement of facilitators or therapists, which were part of the intervention.

1a-iii) Primary condition or target group in the title

Mention primary condition or target group in the title, if any (e.g., “for children with Type I Diabetes”) Example: A Web-based and Mobile Intervention with Telephone Support for Children with Type I Diabetes: Randomized Controlled Trial

	1	2	3	4	5	
subitem not at all important	○	○	○	○	◉	essential

선택해제

Does your paper address subitem 1a-iii? *

Copy and paste relevant sections from manuscript title (include quotes in quotation marks "like this" to indicate direct quotes from your manuscript), or elaborate on this item by providing additional information not in the ms, or briefly explain why the item is not applicable/relevant for your study

Yes, the paper addresses subitem 1a-iii. The manuscript title clearly identifies the primary condition and target group:
"Efficacy of Virtual Reality-Based Biofeedback on Sleep Quality in Individuals with Depression or Anxiety Symptoms: A Randomized Controlled Trial."
In this title, the primary condition (depression or anxiety symptoms) and target group (individuals experiencing these symptoms) are explicitly mentioned. This ensures clarity and specificity, aligning with the guidance for addressing the primary condition and target population in the title.

1b) ABSTRACT: Structured summary of trial design, methods, results, and conclusions

NPT extension: Description of experimental treatment, comparator, care providers, centers, and blinding status.

1b-i) Key features/functionalities/components of the intervention and comparator in the METHODS section of the ABSTRACT

Mention key features/functionalities/components of the intervention and comparator in the abstract. If possible, also mention theories and principles used for designing the site. Keep in mind the needs of systematic reviewers and indexers by including important synonyms. (Note: Only report in the abstract what the main paper is reporting. If this information is missing from the main body of text, consider adding it)

	1	2	3	4	5	
subitem not at all important	○	○	○	◉	○	essential

선택해제

Does your paper address subitem 1b-i? *

Copy and paste relevant sections from the manuscript abstract (include quotes in quotation marks "like this" to indicate direct quotes from your manuscript), or elaborate on this item by providing additional information not in the ms, or briefly explain why the item is not applicable/relevant for your study

"Participants with depressive or anxiety symptoms were randomly assigned to receive either VR-based biofeedback, which integrates immersive virtual reality technology to enhance relaxation and provide physiological feedback, or conventional biofeedback using standard physiological monitoring devices. The primary outcome, sleep quality, was assessed using PSQI scores before and after the 4-week intervention."
This description highlights the essential functionalities of the intervention (VR-based biofeedback) and comparator (conventional biofeedback) and is included in the Methods section of the abstract.

1b-ii) Level of human involvement in the METHODS section of the ABSTRACT

Clarify the level of human involvement in the abstract, e.g., use phrases like “fully automated” vs. “therapist/nurse/care provider/physician-assisted” (mention number and expertise of providers involved, if any). (Note: Only report in the abstract what the main paper is reporting. If this information is missing from the main body of text, consider adding it)

	1	2	3	4	5	
subitem not at all important	○	○	○	○	◉	essential

선택해제

Does your paper address subitem 1b-ii?

Copy and paste relevant sections from the manuscript abstract (include quotes in quotation marks "like this" to indicate direct quotes from your manuscript), or elaborate on this item by providing additional information not in the ms, or briefly explain why the item is not applicable/relevant for your study

"All participants attended sessions at weeks 0, 2, and 4, completing assessments including the Montgomery-Asberg Depression Rating Scale (MADRS), State-Trait Anxiety Inventory (STAI), Visual Analog Scale (VAS) with a therapist."

1b-iii) Open vs. closed, web-based (self-assessment) vs. face-to-face assessments in the METHODS section of the ABSTRACT

Mention how participants were recruited (online vs. offline), e.g., from an open access website or from a clinic or a closed online user group (closed usergroup trial), and clarify if this was a purely web-based trial, or there were face-to-face components (as part of the intervention or for assessment). Clearly say if outcomes were self-assessed through questionnaires (as common in web-based trials). Note: In traditional offline trials, an open trial (open-label trial) is a type of clinical trial in which both the researchers and participants know which treatment is being administered. To avoid confusion, use “blinded” or “unblinded” to indicated the level of blinding instead of “open”, as “open” in web-based trials usually refers to “open access” (i.e. participants can self-enrol). (Note: Only report in the abstract what the main paper is reporting. If this information is missing from the main body of text, consider adding it)

	1	2	3	4	5	
subitem not at all important	○	○	○	○	◉	essential

선택해제

Does your paper address subitem 1b-iii?

Copy and paste relevant sections from the manuscript abstract (include quotes in quotation marks "like this" to indicate direct quotes from your manuscript), or elaborate on this item by providing additional information not in the ms, or briefly explain why the item is not applicable/relevant for your study

"Participants scoring ≥10 on the PHQ-9 or ≥9 on the PDSS were allocated to the depressive and anxiety symptoms (DAS) group, while others were assigned to the healthy control (HC) group. The DAS group was subsequently randomized into VR-based BF or conventional BF interventions. All participants attended sessions at weeks 0, 2, and 4, completing assessments including the Montgomery-Asberg Depression Rating Scale (MADRS), State-Trait Anxiety Inventory (STAI), Visual Analog Scale (VAS) with a therapist. The PSQI was administered at baseline and post-intervention to evaluate alterations in sleep quality over the 4-week period."

1b-iv) RESULTS section in abstract must contain use data

Report number of participants enrolled/assessed in each group, the use/uptake of the intervention (e.g., attrition/adherence metrics, use over time, number of logins etc.), in addition to primary/secondary outcomes. (Note: Only report in the abstract what the main paper is reporting. If this information is missing from the main body of text, consider adding it)

	1	2	3	4	5	
subitem not at all important	○	○	○	○	◉	essential

선택해제

Does your paper address subitem 1b-iv?

Copy and paste relevant sections from the manuscript abstract (include quotes in quotation marks "like this" to indicate direct quotes from your manuscript), or elaborate on this item by providing additional information not in the ms, or briefly explain why the item is not applicable/relevant for your study

"Both DAS/VR and DAS/BF showed significant sleep disturbance improvements, with reductions of -0.58 (SD: 0.75) and -0.66 (SD: 0.75). The DAS/VR had a greater improvement in sleep disturbance compared to HC/VR (P=0.001). The Global PSQI Score improved in both DAS/VR and DAS/BF, decreasing by -2.5 (SD: 2.89) and -3.39 (SD: 2.80) with DAS/VR showing significant improvement compared to HC/VR (P=0.012)."

1b-v) CONCLUSIONS/DISCUSSION in abstract for negative trials

Conclusions/Discussions in abstract for negative trials: Discuss the primary outcome - if the trial is negative (primary outcome not changed), and the intervention was not used, discuss whether negative results are attributable to lack of uptake and discuss reasons. (Note: Only report in the abstract what the main paper is reporting. If this information is missing from the main body of text, consider adding it)

	1	2	3	4	5	
subitem not at all important	○	○	○	○	◉	essential

선택해제

Does your paper address subitem 1b-v?

Copy and paste relevant sections from the manuscript abstract (include quotes in quotation marks "like this" to indicate direct quotes from your manuscript), or elaborate on this item by providing additional information not in the ms, or briefly explain why the item is not applicable/relevant for your study

"This study provides evidence that VR-based biofeedback is an efficacious psychological intervention for enhancing sleep quality in individuals with depressive or anxiety symptoms. These findings suggest potential applications of VR-based biofeedback in clinical settings to improve sleep quality and mental well-being. However, the lack of objective sleep measures and the short duration of the intervention are limitations that warrant further investigation in future studies. Expanding this research with larger and more diverse populations could help validate and generalize these results."

INTRODUCTION

2a) In INTRODUCTION: Scientific background and explanation of rationale

2a-i) Problem and the type of system/solution

Describe the problem and the type of system/solution that is object of the study: intended as stand-alone intervention vs. incorporated in broader health care program? Intended for a particular patient population? Goals of the intervention, e.g., being more cost-effective to other interventions, replace or complement other solutions? (Note: Details about the intervention are provided in “Methods” under 5)

	1	2	3	4	5	
subitem not at all important	○	○	○	○	◉	essential

선택해제

Does your paper address subitem 2a-i? *

Copy and paste relevant sections from the manuscript (include quotes in quotation marks "like this" to indicate direct quotes from your manuscript), or elaborate on this item by providing additional information not in the ms, or briefly explain why the item is not applicable/relevant for your study

"Virtual reality (VR) technologies can give users the capacity to create and manage virtual interactive environments that can be utilized to alter their physiological and emotional responses." "VR operates by deflecting attention and submerging users in soothing simulations designed to activate and regulate different physiological and mental functions that may induce sleep."

2a-ii) Scientific background, rationale: What is known about the (type of) system

Scientific background, rationale: What is known about the (type of) system that is the object of the study (be sure to discuss the use of similar systems for other conditions/diagnoses, if appropiate), motivation for the study, i.e. what are the reasons for and what is the context for this specific study, from which stakeholder viewpoint is the study performed, potential impact of findings [2]. Briefly justify the choice of the comparator.

	1	2	3	4	5	
subitem not at all important	○	○	○	○	◉	essential

선택해제

Does your paper address subitem 2a-ii? *

Copy and paste relevant sections from the manuscript (include quotes in quotation marks "like this" to indicate direct quotes from your manuscript), or elaborate on this item by providing additional information not in the ms, or briefly explain why the item is not applicable/relevant for your study

"HRV-BF has shown effectiveness in reducing stress, anxiety, and depressive symptoms while improving psychological well-being and sleep quality" "Virtual reality (VR) technologies can give users the capacity to create and manage virtual interactive environments that can be utilized to alter their physiological and emotional responses. Consequently, virtual natural environments appear to constitute a suitable backdrop for the application of BF as they can effectively redirect attentional resources in a calming and tranquil manner while affording numerous possibilities for immersive and contextually pertinent feedback elements."

2b) In INTRODUCTION: Specific objectives or hypotheses

Does your paper address CONSORT subitem 2b? *

Copy and paste relevant sections from the manuscript (include quotes in quotation marks "like this" to indicate direct quotes from your manuscript), or elaborate on this item by providing additional information not in the ms, or briefly explain why the item is not applicable/relevant for your study

"Our hypothesis was that VR-based BF could enhance sleep quality among individuals exhibiting symptoms of depression and anxiety. The primary objective of this study was to examine alterations in sleep quality at 4-week follow-up assessment after implementing VR-based BF intervention compared to the sleep quality at baseline."

METHODS

3a) Description of trial design (such as parallel, factorial) including allocation ratio

Does your paper address CONSORT subitem 3a? *

Copy and paste relevant sections from the manuscript (include quotes in quotation marks "like this" to indicate direct quotes from your manuscript), or elaborate on this item by providing additional information not in the ms, or briefly explain why the item is not applicable/relevant for your study

This study was conducted as a 4-week randomized controlled study at a single medical center, in which the efficacy of VR-based BF was compared to that of conventional BF with a therapist. Participants with depressive and anxiety symptoms were randomly assigned to either the VR-based BF (DAS/VR) or conventional BF (DAS/BF) at a 1:1 ratio using computer-generated randomized numbers. Randomization was conducted by an independent researcher not involved in participant recruitment or outcome assessments, ensuring allocation concealment. This process was implemented to minimize selection bias and maintain the integrity of the randomization.

3b) Important changes to methods after trial commencement (such as eligibility criteria), with reasons

Does your paper address CONSORT subitem 3b? *

Copy and paste relevant sections from the manuscript (include quotes in quotation marks "like this" to indicate direct quotes from your manuscript), or elaborate on this item by providing additional information not in the ms, or briefly explain why the item is not applicable/relevant for your study

"this investigation adhered strictly to the registered protocol, and no deviations occurred during its implementation."
No important changes were made to the study methods, including eligibility criteria, trial design, or outcome assessments, after the trial commenced. The study was conducted as originally planned and outlined in the registered protocol to maintain the integrity and validity of the results.

3b-i) Bug fixes, Downtimes, Content Changes

Bug fixes, Downtimes, Content Changes: ehealth systems are often dynamic systems. A description of changes to methods therefore also includes important changes made on the intervention or comparator during the trial (e.g., major bug fixes or changes in the functionality or content) (5-iii) and other "unexpected events" that may have influenced study design such as staff changes, system failures/downtimes, etc. [2].

	1	2	3	4	5	
subitem not at all important	◉	○	○	○	○	essential

선택해제

Does your paper address subitem 3b-i?

Copy and paste relevant sections from the manuscript (include quotes in quotation marks "like this" to indicate direct quotes from your manuscript), or elaborate on this item by providing additional information not in the ms, or briefly explain why the item is not applicable/relevant for your study

"this investigation adhered strictly to the registered protocol, and no deviations occurred during its implementation."
No major bug fixes, system downtimes, or changes to the VR-based BF content occurred during the trial. The VR equipment and software functioned as expected throughout the study, and no unexpected events such as staff changes or system failures impacted the trial's design or execution. This ensured the consistency and reliability of the intervention and data collection processes.

4a) Eligibility criteria for participants

Does your paper address CONSORT subitem 4a? *

Copy and paste relevant sections from the manuscript (include quotes in quotation marks "like this" to indicate direct quotes from your manuscript), or elaborate on this item by providing additional information not in the ms, or briefly explain why the item is not applicable/relevant for your study

"The screening process involved the use of the Korean version of the Mini International Neuropsychiatric Interview (MINI) to comply with the Diagnostic and Statistical Manual of Mental Disorders (DSM-IV). This study had two cohorts: 1) a depressive and anxiety symptom (DAS) group, and 2) a healthy control (HC) group. Inclusion criteria for the DAS group were as follows: self-reported subjective anxiety or depression, no psychiatric treatment within the past six months, and scored ≥ 10 on the Patient Health Questionnaire-9 (PHQ-9) [45] or ≥ 9 on the Panic Disorder Severity Scale (PDSS).

4a-i) Computer / Internet literacy

Computer / Internet literacy is often an implicit “de facto” eligibility criterion - this should be explicitly clarified.

	1	2	3	4	5	
subitem not at all important	◉	○	○	○	○	essential

선택해제

Does your paper address subitem 4a-i?

Copy and paste relevant sections from the manuscript (include quotes in quotation marks "like this" to indicate direct quotes from your manuscript), or elaborate on this item by providing additional information not in the ms, or briefly explain why the item is not applicable/relevant for your study

Participants in this study were required to use VR-based biofeedback technology, which may imply a basic level of comfort with electronic devices. However, no explicit assessment or inclusion/exclusion criterion regarding computer or internet literacy was outlined in the manuscript. Since the VR-based intervention involved direct guidance from a therapist, it was assumed that participants would receive sufficient instructions to overcome any technological barriers.

4a-ii) Open vs. closed, web-based vs. face-to-face assessments:

Open vs. closed, web-based vs. face-to-face assessments: Mention how participants were recruited (online vs. offline), e.g., from an open access website or from a clinic, and clarify if this was a purely web-based trial, or there were face-to-face components (as part of the intervention or for assessment), i.e., to what degree got the study team to know the participant. In online-only trials, clarify if participants were quasi-anonymous and whether having multiple identities was possible or whether technical or logistical measures (e.g., cookies, email confirmation, phone calls) were used to detect/prevent these.

	1	2	3	4	5	
subitem not at all important	○	○	○	○	◉	essential

선택해제

Does your paper address subitem 4a-ii? *

Copy and paste relevant sections from the manuscript (include quotes in quotation marks "like this" to indicate direct quotes from your manuscript), or elaborate on this item by providing additional information not in the ms, or briefly explain why the item is not applicable/relevant for your study

"All participants were asked to visit the Clinical Study Center at Samsung Medical Center three times (at weeks 0, 2, and 4) to receive the allocated intervention, followed by interviews to assess the Montgomery-Asberg Depression Rating Scale (MADRS) [48], the State-Trait Anxiety Inventory (STAI), and the visual analog scale (VAS) after each session. Although participants visited the Clinical Study Center in weeks 0, 2, and 4, the PSQI was specifically administered at the baseline and post-intervention time points to evaluate the overall change in sleep quality over the 4-week intervention period."

4a-iii) Information giving during recruitment

Information given during recruitment. Specify how participants were briefed for recruitment and in the informed consent procedures (e.g., publish the informed consent documentation as appendix, see also item X26), as this information may have an effect on user self-selection, user expectation and may also bias results.

	1	2	3	4	5	
subitem not at all important	○	○	○	○	◉	essential

선택해제

Does your paper address subitem 4a-iii?

Copy and paste relevant sections from the manuscript (include quotes in quotation marks "like this" to indicate direct quotes from your manuscript), or elaborate on this item by providing additional information not in the ms, or briefly explain why the item is not applicable/relevant for your study

All eligible participants provided written informed consent prior to their inclusion in this study.

4b) Settings and locations where the data were collected

Does your paper address CONSORT subitem 4b? *

Copy and paste relevant sections from the manuscript (include quotes in quotation marks "like this" to indicate direct quotes from your manuscript), or elaborate on this item by providing additional information not in the ms, or briefly explain why the item is not applicable/relevant for your study

"All participants were asked to visit the Clinical Study Center at Samsung Medical Center three times (at weeks 0, 2, and 4) to receive the allocated intervention, followed by interviews to assess the Montgomery-Asberg Depression Rating Scale (MADRS), the State-Trait Anxiety Inventory (STAI), and the visual analog scale (VAS) after each session."

4b-i) Report if outcomes were (self-)assessed through online questionnaires

Clearly report if outcomes were (self-)assessed through online questionnaires (as common in web-based trials) or otherwise.

	1	2	3	4	5	
subitem not at all important	(●)	○	○	○	○	essential

선택해제

Does your paper address subitem 4b-i? *

Copy and paste relevant sections from the manuscript (include quotes in quotation marks "like this" to indicate direct quotes from your manuscript), or elaborate on this item by providing additional information not in the ms, or briefly explain why the item is not applicable/relevant for your study

The paper does not report any online or self-assessed outcomes. Instead, all outcomes were assessed face-to-face during clinical visits at the Clinical Study Center of Samsung Medical Center, as described in the manuscript.

4b-ii) Report how institutional affiliations are displayed

Report how institutional affiliations are displayed to potential participants [on ehealth media], as affiliations with prestigious hospitals or universities may affect volunteer rates, use, and reactions with regards to an intervention.(Not a required item – describe only if this may bias results)

	1	2	3	4	5	
subitem not at all important	○	○	○	◉	○	essential

선택해제

Does your paper address subitem 4b-ii?

Copy and paste relevant sections from the manuscript (include quotes in quotation marks "like this" to indicate direct quotes from your manuscript), or elaborate on this item by providing additional information not in the ms, or briefly explain why the item is not applicable/relevant for your study

The manuscript does not explicitly discuss how institutional affiliations were displayed to potential participants. However, recruitment materials stated that the study was conducted at the Samsung Medical Center, a well-known and prestigious institution in South Korea. This affiliation may have influenced participant perceptions of the intervention's credibility and encouraged volunteer participation.
If needed, additional information can be included in the manuscript to clarify how Samsung Medical Center's reputation may have affected recruitment rates or participant expectations. However, this was not deemed a potential source of bias during the study design or recruitment process.

5) The interventions for each group with sufficient details to allow replication, including how and when they were actually administered

5-i) Mention names, credential, affiliations of the developers, sponsors, and owners

Mention names, credential, affiliations of the developers, sponsors, and owners [6] (if authors/evaluators are owners or developer of the software, this needs to be declared in a “Conflict of interest” section or mentioned elsewhere in the manuscript).

	1	2	3	4	5	
subitem not at all important	○	○	○	○	◉	essential

선택해제

Does your paper address subitem 5-i?

Copy and paste relevant sections from the manuscript (include quotes in quotation marks "like this" to indicate direct quotes from your manuscript), or elaborate on this item by providing additional information not in the ms, or briefly explain why the item is not applicable/relevant for your study

"During the VR session, a Samsung Odyssey plus (Samsung Electronics Co., Ltd., Suwon, South Korea) was used with peripheral devices, including the head-mounted display (HMD) with head tracking and stereo earphones, connected. Each participant sat in the motion chair and wore the HMD." This study has no conflicts of interest regarding the VR equipment, and no authors are affiliated with or have financial interests in Samsung Electronics. The VR-based biofeedback software was developed independently for research purposes, and no commercial sponsors or external developers were involved in its creation.

5-ii) Describe the history/development process

Describe the history/development process of the application and previous formative evaluations (e.g., focus groups, usability testing), as these will have an impact on adoption/use rates and help with interpreting results.

	1	2	3	4	5	
subitem not at all important	(●)	( )	( )	( )	( )	essential

선택해제

Does your paper address subitem 5-ii?

Copy and paste relevant sections from the manuscript (include quotes in quotation marks "like this" to indicate direct quotes from your manuscript), or elaborate on this item by providing additional information not in the ms, or briefly explain why the item is not applicable/relevant for your study

The manuscript does not specifically address the history or development process of the VR-based biofeedback application used in this study. However, the intervention design, including the VR content and biofeedback integration, was based on prior research and existing evidence on the effectiveness of HRV-BF and VR-based relaxation interventions. No explicit mention of formative evaluations such as focus groups or usability testing was included in the manuscript.

5-iii) Revisions and updating

Revisions and updating. Clearly mention the date and/or version number of the application/intervention (and comparator, if applicable) evaluated, or describe whether the intervention underwent major changes during the evaluation process, or whether the development and/or content was “frozen” during the trial. Describe dynamic components such as news feeds or changing content which may have an impact on the replicability of the intervention (for unexpected events see item 3b).

	1	2	3	4	5	
subitem not at all important	(●)	( )	( )	( )	( )	essential

선택해제

Does your paper address subitem 5-iii?

Copy and paste relevant sections from the manuscript (include quotes in quotation marks "like this" to indicate direct quotes from your manuscript), or elaborate on this item by providing additional information not in the ms, or briefly explain why the item is not applicable/relevant for your study

The manuscript does not explicitly mention revisions or updates to the VR-based biofeedback intervention or comparator during the trial. As such, no major changes to the content, features, or delivery of the intervention were reported during the evaluation process. The intervention design was "frozen" from the commencement of the trial to ensure consistency across all participants.

5-iv) Quality assurance methods

Provide information on quality assurance methods to ensure accuracy and quality of information provided [1], if applicable.

	1	2	3	4	5	
subitem not at all important	○	○	○	○	◉	essential

선택해제

Does your paper address subitem 5-iv?

Copy and paste relevant sections from the manuscript (include quotes in quotation marks "like this" to indicate direct quotes from your manuscript), or elaborate on this item by providing additional information not in the ms, or briefly explain why the item is not applicable/relevant for your study

All participants were asked to visit the Clinical Study Center at Samsung Medical Center three times (at weeks 0, 2, and 4) to receive the allocated intervention, followed by interviews to assess the Montgomery-Asberg Depression Rating Scale (MADRS), the State-Trait Anxiety Inventory (STAI), and the visual analog scale (VAS) after each session. Although participants visited the Clinical Study Center in weeks 0, 2, and 4, the PSQI was specifically administered at the baseline and post-intervention time points to evaluate the overall change in sleep quality over the 4-week intervention period.

5-v) Ensure replicability by publishing the source code, and/or providing screenshots/screen-capture video, and/or providing flowcharts of the algorithms used

Ensure replicability by publishing the source code, and/or providing screenshots/screen-capture video, and/or providing flowcharts of the algorithms used. Replicability (i.e., other researchers should in principle be able to replicate the study) is a hallmark of scientific reporting.

	1	2	3	4	5	
subitem not at all important	◉	○	○	○	○	essential

선택해제

Does your paper address subitem 5-v?

Copy and paste relevant sections from the manuscript (include quotes in quotation marks "like this" to indicate direct quotes from your manuscript), or elaborate on this item by providing additional information not in the ms, or briefly explain why the item is not applicable/relevant for your study

The manuscript does not explicitly address the provision of source code, screenshots, screen-capture videos, or flowcharts of the algorithms used for VR-based biofeedback intervention.

5-vi) Digital preservation

Digital preservation: Provide the URL of the application, but as the intervention is likely to change or disappear over the course of the years; also make sure the intervention is archived (Internet Archive, webcitation.org, and/or publishing the source code or screenshots/videos alongside the article). As pages behind login screens cannot be archived, consider creating demo pages which are accessible without login.

	1	2	3	4	5	
subitem not at all important	◉	○	○	○	○	essential

선택해제

Does your paper address subitem 5-vi?

Copy and paste relevant sections from the manuscript (include quotes in quotation marks "like this" to indicate direct quotes from your manuscript), or elaborate on this item by providing additional information not in the ms, or briefly explain why the item is not applicable/relevant for your study

The manuscript does not explicitly address digital preservation, such as providing the URL for the VR-based biofeedback application or archiving the intervention on platforms like Internet Archive or Webcitation.org. Additionally, no source code, screenshots, or demo pages of the intervention are included in the manuscript.

5-vii) Access

Access: Describe how participants accessed the application, in what setting/context, if they had to pay (or were paid) or not, whether they had to be a member of specific group. If known, describe how participants obtained “access to the platform and Internet” [1]. To ensure access for editors/reviewers/readers, consider to provide a “backdoor” login account or demo mode for reviewers/readers to explore the application (also important for archiving purposes, see vi).

	1	2	3	4	5	
subitem not at all important	○	○	◉	○	○	essential

선택해제

Does your paper address subitem 5-vii? *

Copy and paste relevant sections from the manuscript (include quotes in quotation marks "like this" to indicate direct quotes from your manuscript), or elaborate on this item by providing additional information not in the ms, or briefly explain why the item is not applicable/relevant for your study

The manuscript partially addresses the access to the intervention by providing details about the setting in which the intervention was administered. Specifically, participants accessed the VR-based biofeedback system and conventional biofeedback intervention during their scheduled visits to the Clinical Study Center at Samsung Medical Center. Access to the intervention was free of charge, and participants were compensated for their time and participation.

5-viii) Mode of delivery, features/functionalities/components of the intervention and comparator, and the theoretical framework

Describe mode of delivery, features/functionalities/components of the intervention and comparator, and the theoretical framework [6] used to design them (instructional strategy [1], behaviour change techniques, persuasive features, etc., see e.g., [7, 8] for terminology). This includes an in-depth description of the content (including where it is coming from and who developed it) [1]," whether [and how] it is tailored to individual circumstances and allows users to track their progress and receive feedback" [6]. This also includes a description of communication delivery channels and – if computer-mediated communication is a component – whether communication was synchronous or asynchronous [6]. It also includes information on presentation strategies [1], including page design principles, average amount of text on pages, presence of hyperlinks to other resources, etc. [1].

	1	2	3	4	5	
subitem not at all important	○	○	○	◉	○	essential

선택해제

Does your paper address subitem 5-viii? *

Copy and paste relevant sections from the manuscript (include quotes in quotation marks "like this" to indicate direct quotes from your manuscript), or elaborate on this item by providing additional information not in the ms, or briefly explain why the item is not applicable/relevant for your study

"Participants watched and listened to VR relaxation training video comprised of four natural scenes for five minutes. A psychiatrist (HJJ) led participants through a breathing exercise during the VR session. After an ancient bell rang, participants started to slowly wander through nature in virtual reality while listening to classically soothing background music and digital nature sounds that were specifically designed to go with VR images, such as sounds of birds chirping, wind, water flowing, and rustling leaves, specifically designed to accompany the VR images. At the same time, guided relaxation therapy was performed (such as, "Relax your muscles. Breathe in slowly while your stomach expands, then exhale one, two, three times. Exhale gradually as your abdomen expands"). While practicing relaxation techniques, participants were permitted to slowly cross a river, soar to skies, and stroll through a tranquil meadow in the VR."

5-ix) Describe use parameters

Describe use parameters (e.g., intended “doses” and optimal timing for use). Clarify what instructions or recommendations were given to the user, e.g., regarding timing, frequency, heaviness of use, if any, or was the intervention used ad libitum.

	1	2	3	4	5	
subitem not at all important	○	○	○	◉	○	essential

선택해제

Does your paper address subitem 5-ix?

Copy and paste relevant sections from the manuscript (include quotes in quotation marks "like this" to indicate direct quotes from your manuscript), or elaborate on this item by providing additional information not in the ms, or briefly explain why the item is not applicable/relevant for your study

"All participants were asked to visit the Clinical Study Center at Samsung Medical Center three times (at weeks 0, 2, and 4) to receive the allocated intervention."

5-x) Clarify the level of human involvement

Clarify the level of human involvement (care providers or health professionals, also technical assistance) in the e-intervention or as co-intervention (detail number and expertise of professionals involved, if any, as well as “type of assistance offered, the timing and frequency of the support, how it is initiated, and the medium by which the assistance is delivered”. It may be necessary to distinguish between the level of human involvement required for the trial, and the level of human involvement required for a routine application outside of a RCT setting (discuss under item 21 – generalizability).

	1	2	3	4	5	
subitem not at all important	○	○	○	◉	○	essential

선택해제

Does your paper address subitem 5-x?

Copy and paste relevant sections from the manuscript (include quotes in quotation marks "like this" to indicate direct quotes from your manuscript), or elaborate on this item by providing additional information not in the ms, or briefly explain why the item is not applicable/relevant for your study

A psychiatrist (HJJ) led participants through a breathing exercise during the VR session.

5-xi) Report any prompts/reminders used

Report any prompts/reminders used: Clarify if there were prompts (letters, emails, phone calls, SMS) to use the application, what triggered them, frequency etc. It may be necessary to distinguish between the level of prompts/reminders required for the trial, and the level of prompts/reminders for a routine application outside of a RCT setting (discuss under item 21 – generalizability).

	1	2	3	4	5	
subitem not at all important	○	◉	○	○	○	essential

선택해제

Does your paper address subitem 5-xi? *

Copy and paste relevant sections from the manuscript (include quotes in quotation marks "like this" to indicate direct quotes from your manuscript), or elaborate on this item by providing additional information not in the ms, or briefly explain why the item is not applicable/relevant for your study

The manuscript does not specifically mention the use of prompts or reminders, such as emails, phone calls, or SMS, to encourage participants to use the intervention or complete their follow-up visits. All participants visited the Clinical Study Center in person for their scheduled sessions, and adherence was monitored during these visits. Since this study involved in-person sessions under the direct supervision of professionals, additional prompts or reminders were not deemed necessary.

5-xii) Describe any co-interventions (incl. training/support)

Describe any co-interventions (incl. training/support): Clearly state any interventions that are provided in addition to the targeted eHealth intervention, as ehealth intervention may not be designed as stand-alone intervention. This includes training sessions and support [1]. It may be necessary to distinguish between the level of training required for the trial, and the level of training for a routine application outside of a RCT setting (discuss under item 21 – generalizability.

	1	2	3	4	5	
subitem not at all important	○	◉	○	○	○	essential

선택해제

Does your paper address subitem 5-xii? *

Copy and paste relevant sections from the manuscript (include quotes in quotation marks "like this" to indicate direct quotes from your manuscript), or elaborate on this item by providing additional information not in the ms, or briefly explain why the item is not applicable/relevant for your study

The manuscript includes a description of the primary intervention but does not mention any additional co-interventions, such as separate training sessions or additional support. As described in the Methods section:
"A psychiatrist (HJJ) led participants through a breathing exercise during the VR session."
"Regarding conventional BF, a computerized biofeedback device ProComp Infiniti (Thought Technology, Ltd., Montreal, Canada) was used. Participants were instructed about relaxation techniques while observing physiological parameter signals, which encompassed heart rate/blood pressure (HR/BP), skin conductance, respiration, and skin temperature displayed on the screen with a therapist. During the BF session, the researcher provided feedback to subjects when physiological markers changed by 15%. Five minutes were spent carrying out the BF intervention."

6a) Completely defined pre-specified primary and secondary outcome measures, including how and when they were assessed

Does your paper address CONSORT subitem 6a? *

Copy and paste relevant sections from the manuscript (include quotes in quotation marks "like this" to indicate direct quotes from your manuscript), or elaborate on this item by providing additional information not in the ms, or briefly explain why the item is not applicable/relevant for your study

"the PSQI was specifically administered at the baseline and post-intervention time points to evaluate the overall change in sleep quality over the 4-week intervention period."
"Participants were requested to recollect and report their sleep patterns from the preceding month, responding to a set of 19 distinct inquiries related to seven primary components of sleep, including overall sleep quality, sleep latency, sleep duration, sleep efficiency, sleep disturbance, sleep medicine uses, and day dysfunction due to sleepiness using the PSQI.
The PSQI was administered at baseline (week 0) and post-intervention (week 4) to evaluate."

6a-i) Online questionnaires: describe if they were validated for online use and apply CHERRIES items to describe how the questionnaires were designed/deployed

If outcomes were obtained through online questionnaires, describe if they were validated for online use and apply CHERRIES items to describe how the questionnaires were designed/deployed [9].

	1	2	3	4	5	
subitem not at all important	◉	○	○	○	○	essential

선택해제

Does your paper address subitem 6a-i?

Copy and paste relevant sections from manuscript text

Because no online questionnaires were used, validation for online use and application of CHERRIES (Checklist for Reporting Results of Internet E-Surveys) guidelines are not applicable to this study.

6a-ii) Describe whether and how “use” (including intensity of use/dosage) was defined/measured/monitored

Describe whether and how “use” (including intensity of use/dosage) was defined/measured/monitored (logins, logfile analysis, etc.). Use/adoption metrics are important process outcomes that should be reported in any ehealth trial.

	1	2	3	4	5	
subitem not at all important	○	◉	○	○	○	essential

선택해제

Does your paper address subitem 6a-ii?

Copy and paste relevant sections from manuscript text

The study clearly defined and administered the intervention during scheduled visits, but it did not include mechanisms to measure the intensity of use or adherence outside these sessions (e.g., home practice). Therefore, detailed use/adoption metrics (such as logins or logfile analysis) were not applicable to the trial design.

6a-iii) Describe whether, how, and when qualitative feedback from participants was obtained

Describe whether, how, and when qualitative feedback from participants was obtained (e.g., through emails, feedback forms, interviews, focus groups).

	1	2	3	4	5	
subitem not at all important	○	◉	○	○	○	essential

선택해제

Does your paper address subitem 6a-iii?

Copy and paste relevant sections from manuscript text

The manuscript does not specifically address qualitative feedback from participants (e.g., through emails, feedback forms, interviews, or focus groups). The study primarily focused on quantitative outcome measures, including PSQI, MADRS, STAI, and VAS scores, to assess the efficacy of the intervention.

6b) Any changes to trial outcomes after the trial commenced, with reasons

Does your paper address CONSORT subitem 6b? *

Copy and paste relevant sections from the manuscript (include quotes in quotation marks "like this" to indicate direct quotes from your manuscript), or elaborate on this item by providing additional information not in the ms, or briefly explain why the item is not applicable/relevant for your study

"this investigation adhered strictly to the registered protocol, and no deviations occurred during its implementation."

7a) How sample size was determined

NPT: When applicable, details of whether and how the clustering by care provides or centers was addressed

7a-i) Describe whether and how expected attrition was taken into account when calculating the sample size

Describe whether and how expected attrition was taken into account when calculating the sample size.

	1	2	3	4	5	
subitem not at all important	○	○	○	○	●	essential

선택해제

Does your paper address subitem 7a-i?

Copy and paste relevant sections from manuscript title (include quotes in quotation marks "like this" to indicate direct quotes from your manuscript), or elaborate on this item by providing additional information not in the ms, or briefly explain why the item is not applicable/relevant for your study

"The sample size for this study was determined based on a previous study, which reported an average effect size of 0.69 for all items in the MADRS. The primary goal of this study was to evaluate the effect size difference between VR-based BF and conventional BF in participants with DAS. Assuming a moderate-level effect size of 0.7 in MADRS, a minimum of 34 participants per group was required to achieve a type I error rate of 0.05 and a statistical power of 80%. To account for an anticipated dropout rate of 15%, a total of 120 participants were planned for recruitment, comprising 80 participants in the DAS cohort and 40 participants in the HC cohort."

7b) When applicable, explanation of any interim analyses and stopping guidelines

Does your paper address CONSORT subitem 7b? *

Copy and paste relevant sections from the manuscript (include quotes in quotation marks "like this" to indicate direct quotes from your manuscript), or elaborate on this item by providing additional information not in the ms, or briefly explain why the item is not applicable/relevant for your study

This study did not incorporate interim analyses or stopping guidelines, as it was not a requirement for the trial design. The study adhered to the original protocol without modifications or interruptions.

8a) Method used to generate the random allocation sequence

NPT: When applicable, how care providers were allocated to each trial group

Does your paper address CONSORT subitem 8a? *

Copy and paste relevant sections from the manuscript (include quotes in quotation marks "like this" to indicate direct quotes from your manuscript), or elaborate on this item by providing additional information not in the ms, or briefly explain why the item is not applicable/relevant for your study

Participants with depressive and anxiety symptoms were randomly assigned to either the VR-based BF (DAS/VR) or conventional BF (DAS/BF) at a 1:1 ratio using computer-generated randomized numbers. Randomization was conducted by an independent researcher not involved in participant recruitment or outcome assessments, ensuring allocation concealment. This process was implemented to minimize selection bias and maintain the integrity of the randomization.

8b) Type of randomisation; details of any restriction (such as blocking and block size)

Does your paper address CONSORT subitem 8b? *

Copy and paste relevant sections from the manuscript (include quotes in quotation marks "like this" to indicate direct quotes from your manuscript), or elaborate on this item by providing additional information not in the ms, or briefly explain why the item is not applicable/relevant for your study

"Participants with depressive and anxiety symptoms were randomly assigned to either the VR-based BF (DAS/VR) or conventional BF (DAS/BF) at a 1:1 ratio using computer-generated randomized numbers. Randomization was conducted by an independent researcher not involved in participant recruitment or outcome assessments, ensuring allocation concealment. This process was implemented to minimize selection bias and maintain the integrity of the randomization."

9) Mechanism used to implement the random allocation sequence (such as sequentially numbered containers), describing any steps taken to conceal the sequence until interventions were assigned

Does your paper address CONSORT subitem 9? *

Copy and paste relevant sections from the manuscript (include quotes in quotation marks "like this" to indicate direct quotes from your manuscript), or elaborate on this item by providing additional information not in the ms, or briefly explain why the item is not applicable/relevant for your study

"Participants with depressive and anxiety symptoms were randomly assigned to either the VR-based BF (DAS/VR) or conventional BF (DAS/BF) at a 1:1 ratio using computer-generated randomized numbers."

10) Who generated the random allocation sequence, who enrolled participants, and who assigned participants to interventions

Does your paper address CONSORT subitem 10? *

Copy and paste relevant sections from the manuscript (include quotes in quotation marks "like this" to indicate direct quotes from your manuscript), or elaborate on this item by providing additional information not in the ms, or briefly explain why the item is not applicable/relevant for your study

"Randomization was conducted by an independent researcher not involved in participant recruitment or outcome assessments, ensuring allocation concealment."

11a) If done, who was blinded after assignment to interventions (for example, participants, care providers, those assessing outcomes) and how

NPT: Whether or not administering co-interventions were blinded to group assignment

11a-i) Specify who was blinded, and who wasn’t

Specify who was blinded, and who wasn’t. Usually, in web-based trials it is not possible to blind the participants [1, 3] (this should be clearly acknowledged), but it may be possible to blind outcome assessors, those doing data analysis or those administering co-interventions (if any).

	1	2	3	4	5	
subitem not at all important	○	○	○	◉	○	essential

선택해제

Does your paper address subitem 11a-i? *

Copy and paste relevant sections from the manuscript (include quotes in quotation marks "like this" to indicate direct quotes from your manuscript), or elaborate on this item by providing additional information not in the ms, or briefly explain why the item is not applicable/relevant for your study

"Randomization was conducted by an independent researcher not involved in participant recruitment or outcome assessments, ensuring allocation concealment."

11a-ii) Discuss e.g., whether participants knew which intervention was the “intervention of interest” and which one was the “comparator”

Informed consent procedures (4a-ii) can create biases and certain expectations - discuss e.g., whether participants knew which intervention was the “intervention of interest” and which one was the “comparator”.

	1	2	3	4	5	
subitem not at all important	○	○	○	◉	○	essential

선택해제

Does your paper address subitem 11a-ii?

Copy and paste relevant sections from the manuscript (include quotes in quotation marks "like this" to indicate direct quotes from your manuscript), or elaborate on this item by providing additional information not in the ms, or briefly explain why the item is not applicable/relevant for your study

Randomization was conducted by an independent researcher not involved in participant recruitment or outcome assessments, ensuring allocation concealment.

11b) If relevant, description of the similarity of interventions

(this item is usually not relevant for ehealth trials as it refers to similarity of a placebo or sham intervention to a active medication/intervention)

Does your paper address CONSORT subitem 11b? *

Copy and paste relevant sections from the manuscript (include quotes in quotation marks "like this" to indicate direct quotes from your manuscript), or elaborate on this item by providing additional information not in the ms, or briefly explain why the item is not applicable/relevant for your study

"the efficacy of VR-based BF was compared to that of conventional BF with a therapist."
"In order to evaluate the effect of VR-based BF on healthy subjects, the HC group received VR-based BF intervention (HC/VR)."

12a) Statistical methods used to compare groups for primary and secondary outcomes

NPT: When applicable, details of whether and how the clustering by care providers or centers was addressed

Does your paper address CONSORT subitem 12a? *

Copy and paste relevant sections from the manuscript (include quotes in quotation marks "like this" to indicate direct quotes from your manuscript), or elaborate on this item by providing additional information not in the ms, or briefly explain why the item is not applicable/relevant for your study

"Group comparisons among three groups were conducted using the chi-square test or Fisher’s exact test for categorical variables. For continuous variables, they were compared using one-way analysis of variance (ANOVA) or Kruskal-Wallis test according to satisfaction of normality assumption. Changes in outcome measurements between baseline (0 week) and 4-week follow-up visit were examined via paired t-test or Wilcoxon signed rank test in each group. For changes in outcome measurements, Wilcoxon rank sum test or t-test were used for two pairwise comparisons between VR and BF in DAS and between DAS/VR and HC/VR. After adjusting for sex and age, we additionally performed linear regression analysis to compare between groups. Statistical significance was declared when p-value was less than 0.05. All statistical analyses were done with SAS version 9.4 (SAS Institute, Cary, NC, USA)."

12a-i) Imputation techniques to deal with attrition / missing values

Imputation techniques to deal with attrition / missing values: Not all participants will use the intervention/comparator as intended and attrition is typically high in ehealth trials. Specify how participants who did not use the application or dropped out from the trial were treated in the statistical analysis (a complete case analysis is strongly discouraged, and simple imputation techniques such as LOCF may also be problematic [4]).

	1	2	3	4	5	
subitem not at all important	○	○	○	●	○	essential

선택해제

Does your paper address subitem 12a-i? *

Copy and paste relevant sections from the manuscript (include quotes in quotation marks "like this" to indicate direct quotes from your manuscript), or elaborate on this item by providing additional information not in the ms, or briefly explain why the item is not applicable/relevant for your study

We confirm that there were no missing covariate data in the analysis. Conflict case analysis was conducted to ensure the integrity of the dataset. Furthermore, details regarding participant flow, including dropout cases, have been documented and presented in the CONSORT flow chart for transparency.

12b) Methods for additional analyses, such as subgroup analyses and adjusted analyses

Does your paper address CONSORT subitem 12b? *

Copy and paste relevant sections from the manuscript (include quotes in quotation marks "like this" to indicate direct quotes from your manuscript), or elaborate on this item by providing additional information not in the ms, or briefly explain why the item is not applicable/relevant for your study

"After adjusting for sex and age, we additionally performed linear regression analysis to compare between groups."

X26) REB/IRB Approval and Ethical Considerations [recommended as subheading under "Methods"] (not a CONSORT item)

X26-i) Comment on ethics committee approval

	1	2	3	4	5	
subitem not at all important	○	○	○	○	◉	essential

선택해제

Does your paper address subitem X26-i?

Copy and paste relevant sections from the manuscript (include quotes in quotation marks "like this" to indicate direct quotes from your manuscript), or elaborate on this item by providing additional information not in the ms, or briefly explain why the item is not applicable/relevant for your study

"This study received approval from the Institutional Review Board of Samsung Medical Center (No. SMC 2019-07-039-010)."

x26-ii) Outline informed consent procedures

Outline informed consent procedures e.g., if consent was obtained offline or online (how? Checkbox, etc.?), and what information was provided (see 4a-ii). See [6] for some items to be included in informed consent documents.

	1	2	3	4	5	
subitem not at all important	○	○	○	○	◉	essential

선택해제

Does your paper address subitem X26-ii?

Copy and paste relevant sections from the manuscript (include quotes in quotation marks "like this" to indicate direct quotes from your manuscript), or elaborate on this item by providing additional information not in the ms, or briefly explain why the item is not applicable/relevant for your study

All eligible participants provided written informed consent prior to their inclusion in this study.

X26-iii) Safety and security procedures

Safety and security procedures, incl. privacy considerations, and any steps taken to reduce the likelihood or detection of harm (e.g., education and training, availability of a hotline)

	1	2	3	4	5	
subitem not at all important	○	○	○	○	◉	essential

선택해제

Does your paper address subitem X26-iii?

Copy and paste relevant sections from the manuscript (include quotes in quotation marks "like this" to indicate direct quotes from your manuscript), or elaborate on this item by providing additional information not in the ms, or briefly explain why the item is not applicable/relevant for your study

This study received approval from the Institutional Review Board of Samsung Medical Center (No. SMC 2019-07-039-010). Furthermore, this investigation adhered strictly to the registered protocol, and no deviations occurred during its implementation.

RESULTS

13a) For each group, the numbers of participants who were randomly assigned, received intended treatment, and were analysed for the primary outcome

NPT: The number of care providers or centers performing the intervention in each group and the number of patients treated by each care provider in each center

Does your paper address CONSORT subitem 13a? *

Copy and paste relevant sections from the manuscript (include quotes in quotation marks "like this" to indicate direct quotes from your manuscript), or elaborate on this item by providing additional information not in the ms, or briefly explain why the item is not applicable/relevant for your study

"Between December 2019 and February 2022, a total of 131 adults aged over 18 years self-referred were enrolled in this study through advertising at Samsung Medical Center in Seoul, South Korea. The screening process resulted in exclusion of 13 volunteers due to psychiatric diagnoses, including 9 major depressive disorders, 3 comorbid phobic disorders, and 1 antisocial personality disorder. Finally, 120 participants were enrolled for this study, including 80 participants in the DAS cohort and 40 participants in the HC cohort. Those in the DAS cohort were allocated into the VR intervention group (n=41) and the BF intervention group (n=39) at random. After the first visit, one person in the VR group and one person in the BF group withdrew. Finally, a total of 118 participants were subjected to comprehensive analysis."

13b) For each group, losses and exclusions after randomisation, together with reasons

Does your paper address CONSORT subitem 13b? (NOTE: Preferably, this is shown in a CONSORT flow diagram) *

Copy and paste relevant sections from the manuscript (include quotes in quotation marks "like this" to indicate direct quotes from your manuscript), or elaborate on this item by providing additional information not in the ms, or briefly explain why the item is not applicable/relevant for your study

Between December 2019 and February 2022, a total of 131 adults aged over 18 years self-referred were enrolled in this study through advertising at Samsung Medical Center in Seoul, South Korea. The screening process resulted in exclusion of 13 volunteers due to psychiatric diagnoses, including 9 major depressive disorders, 3 comorbid phobic disorders, and 1 antisocial personality disorder. Finally, 120 participants were enrolled for this study, including 80 participants in the DAS cohort and 40 participants in the HC cohort. Those in the DAS cohort were allocated into the VR intervention group (n=41) and the BF intervention group (n=39) at random. After the first visit, one person in the VR group and one person in the BF group withdrew. Finally, a total of 118 participants were subjected to comprehensive analysis.

13b-i) Attrition diagram

Strongly recommended: An attrition diagram (e.g., proportion of participants still logging in or using the intervention/comparator in each group plotted over time, similar to a survival curve) or other figures or tables demonstrating usage/dose/engagement.

	1	2	3	4	5	
subitem not at all important	○	○	○	●	○	essential

선택해제

Does your paper address subitem 13b-i?

Copy and paste relevant sections from the manuscript or cite the figure number if applicable (include quotes in quotation marks "like this" to indicate direct quotes from your manuscript), or elaborate on this item by providing additional information not in the ms, or briefly explain why the item is not applicable/relevant for your study

Figure 1. CONSORT flow diagram depicting the progression of participants through a clinical trial.

14a) Dates defining the periods of recruitment and follow-up

Does your paper address CONSORT subitem 14a? *

Copy and paste relevant sections from the manuscript (include quotes in quotation marks "like this" to indicate direct quotes from your manuscript), or elaborate on this item by providing additional information not in the ms, or briefly explain why the item is not applicable/relevant for your study

"Between December 2019 and February 2022, a total of 131 adults aged over 18 years self-referred were enrolled in this study through advertising at Samsung Medical Center in Seoul, South Korea. The screening process resulted in exclusion of 13 volunteers due to psychiatric diagnoses, including 9 major depressive disorders, 3 comorbid phobic disorders, and 1 antisocial personality disorder. Finally, 120 participants were enrolled for this study, including 80 participants in the DAS cohort and 40 participants in the HC cohort. Those in the DAS cohort were allocated into the VR intervention group (n=41) and the BF intervention group (n=39) at random. After the first visit, one person in the VR group and one person in the BF group withdrew. Finally, a total of 118 participants were subjected to comprehensive analysis."

14a-i) Indicate if critical “secular events” fell into the study period

Indicate if critical “secular events” fell into the study period, e.g., significant changes in Internet resources available or “changes in computer hardware or Internet delivery resources”

	1	2	3	4	5	
subitem not at all important	○	○	○	●	○	essential

선택해제

Does your paper address subitem 14a-i?

Copy and paste relevant sections from the manuscript (include quotes in quotation marks "like this" to indicate direct quotes from your manuscript), or elaborate on this item by providing additional information not in the ms, or briefly explain why the item is not applicable/relevant for your study

No critical secular events, such as changes in internet resources, hardware, or software, occurred during the study period that could have affected the intervention or data collection process

14b) Why the trial ended or was stopped (early)

Does your paper address CONSORT subitem 14b? *

Copy and paste relevant sections from the manuscript (include quotes in quotation marks "like this" to indicate direct quotes from your manuscript), or elaborate on this item by providing additional information not in the ms, or briefly explain why the item is not applicable/relevant for your study

This trial was completed as planned, with no early termination or deviations from the protocol.

15) A table showing baseline demographic and clinical characteristics for each group

NPT: When applicable, a description of care providers (case volume, qualification, expertise, etc.) and centers (volume) in each group

Does your paper address CONSORT subitem 15? *

Copy and paste relevant sections from the manuscript (include quotes in quotation marks "like this" to indicate direct quotes from your manuscript), or elaborate on this item by providing additional information not in the ms, or briefly explain why the item is not applicable/relevant for your study

Table 1. Baseline characteristics of participants.

15-i) Report demographics associated with digital divide issues

In ehealth trials it is particularly important to report demographics associated with digital divide issues, such as age, education, gender, social-economic status, computer/Internet/ehealth literacy of the participants, if known.

	1	2	3	4	5	
subitem not at all important	○	○	●	○	○	essential

선택해제

Does your paper address subitem 15-i? *

Copy and paste relevant sections from the manuscript (include quotes in quotation marks "like this" to indicate direct quotes from your manuscript), or elaborate on this item by providing additional information not in the ms, or briefly explain why the item is not applicable/relevant for your study

Baseline demographic characteristics, including age, gender, education level, and employment status, were collected and summarized in Table 1 to ensure comparability across groups.

16) For each group, number of participants (denominator) included in each analysis and whether the analysis was by original assigned groups

16-i) Report multiple “denominators” and provide definitions

Report multiple “denominators” and provide definitions: Report N’s (and effect sizes) “across a range of study participation [and use] thresholds” [1], e.g., N exposed, N consented, N used more than x times, N used more than y weeks, N participants “used” the intervention/comparator at specific pre-defined time points of interest (in absolute and relative numbers per group). Always clearly define “use” of the intervention.

	1	2	3	4	5	
subitem not at all important	○	○	○	○	◉	essential

선택해제

Does your paper address subitem 16-i? *

Copy and paste relevant sections from the manuscript (include quotes in quotation marks "like this" to indicate direct quotes from your manuscript), or elaborate on this item by providing additional information not in the ms, or briefly explain why the item is not applicable/relevant for your study

"The screening process resulted in exclusion of 13 volunteers due to psychiatric diagnoses, including 9 major depressive disorders, 3 comorbid phobic disorders, and 1 antisocial personality disorder. Finally, 120 participants were enrolled for this study, including 80 participants in the DAS group and 40 participants in the HC group. Those in the DAS group were allocated into the VR intervention group (n=41) and the BF intervention group (n=39) at random. After the first visit, one person in the VR group and one person in the BF group withdrew. Finally, a total of 118 participants were subjected to comprehensive analysis."

16-ii) Primary analysis should be intent-to-treat

Primary analysis should be intent-to-treat, secondary analyses could include comparing only “users”, with the appropriate caveats that this is no longer a randomized sample (see 18-i).

	1	2	3	4	5	
subitem not at all important	○	○	○	●	○	essential

선택해제

Does your paper address subitem 16-ii?

Copy and paste relevant sections from the manuscript (include quotes in quotation marks "like this" to indicate direct quotes from your manuscript), or elaborate on this item by providing additional information not in the ms, or briefly explain why the item is not applicable/relevant for your study

"The screening process resulted in exclusion of 13 volunteers due to psychiatric diagnoses, including 9 major depressive disorders, 3 comorbid phobic disorders, and 1 antisocial personality disorder. Finally, 120 participants were enrolled for this study, including 80 participants in the DAS group and 40 participants in the HC group. Those in the DAS group were allocated into the VR intervention group (n=41) and the BF intervention group (n=39) at random. After the first visit, one person in the VR group and one person in the BF group withdrew. Finally, a total of 118 participants were subjected to comprehensive analysis."

17a) For each primary and secondary outcome, results for each group, and the estimated effect size and its precision (such as 95% confidence interval)

Does your paper address CONSORT subitem 17a? *

Copy and paste relevant sections from the manuscript (include quotes in quotation marks "like this" to indicate direct quotes from your manuscript), or elaborate on this item by providing additional information not in the ms, or briefly explain why the item is not applicable/relevant for your study

The manuscript provides results for each primary and secondary outcome, including pre- and post-intervention scores and statistical significance (p-values). For example, the global PSQI scores in the DAS/VR group showed a reduction from 9.70 (±2.49) to 7.20 (±2.46) (p<0.001). However, confidence intervals for the estimated effect sizes were not reported in the Original manuscript.

17a-i) Presentation of process outcomes such as metrics of use and intensity of use

In addition to primary/secondary (clinical) outcomes, the presentation of process outcomes such as metrics of use and intensity of use (dose, exposure) and their operational definitions is critical. This does not only refer to metrics of attrition (13-b) (often a binary variable), but also to more continuous exposure metrics such as "average session length". These must be accompanied by a technical description how a metric like a "session" is defined (e.g., timeout after idle time) [1] (report under item 6a).

	1	2	3	4	5	
subitem not at all important	○	○	○	◉	○	essential

선택해제

Does your paper address subitem 17a-i?

Copy and paste relevant sections from the manuscript (include quotes in quotation marks "like this" to indicate direct quotes from your manuscript), or elaborate on this item by providing additional information not in the ms, or briefly explain why the item is not applicable/relevant for your study

Supplementary Table 1. Detailed Timeline and Structure of the VR-Based Biofeedback Intervention Session" provides a breakdown of the session's structure, including metrics of use and intensity. Each VR-based BF session lasted 10 minutes, comprising distinct activities such as a breathing exercise, VR nature immersion, and guided relaxation therapy, with detailed timing for each phase. These process outcomes, including session duration and activity breakdown, allow for a clear understanding of participant exposure to the intervention and provide operational definitions for metrics like session length and intensity of use.

17b) For binary outcomes, presentation of both absolute and relative effect sizes is recommended

Does your paper address CONSORT subitem 17b? *

Copy and paste relevant sections from the manuscript (include quotes in quotation marks "like this" to indicate direct quotes from your manuscript), or elaborate on this item by providing additional information not in the ms, or briefly explain why the item is not applicable/relevant for your study

There are no binary outcomes reported in the study. The primary and secondary outcomes, such as the Pittsburgh Sleep Quality Index (PSQI) scores, Montgomery-Asberg Depression Rating Scale (MADRS) scores, and State-Trait Anxiety Inventory (STAI) scores, are continuous variables. Therefore, absolute and relative effect sizes for binary outcomes are not applicable to this study.

18) Results of any other analyses performed, including subgroup analyses and adjusted analyses, distinguishing pre-specified from exploratory

Does your paper address CONSORT subitem 18? *

Copy and paste relevant sections from the manuscript (include quotes in quotation marks "like this" to indicate direct quotes from your manuscript), or elaborate on this item by providing additional information not in the ms, or briefly explain why the item is not applicable/relevant for your study

"Group comparisons among three groups were conducted using the chi-square test or Fisher's exact test for categorical variables. For continuous variables, they were compared using one-way analysis of variance (ANOVA) or Kruskal-Wallis test according to satisfaction of normality assumption. Changes in outcome measurements between baseline (0 week) and 4-week follow-up visit were examined via paired t-test or Wilcoxon signed rank test in each group. For changes in outcome measurements, Wilcoxon rank sum test or t-test were used for two pairwise comparisons between VR and BF in DAS and between DAS/VR and HC/VR. After adjusting for sex and age, we additionally performed linear regression analysis to compare between groups. Statistical significance was declared when p-value was less than 0.05. All statistical analyses were done with SAS version 9.4 (SAS Institute, Cary, NC, USA)."

18-i) Subgroup analysis of comparing only users

A subgroup analysis of comparing only users is not uncommon in ehealth trials, but if done, it must be stressed that this is a self-selected sample and no longer an unbiased sample from a randomized trial (see 16-iii).

	1	2	3	4	5	
subitem not at all important	○	●	○	○	○	essential

선택해제

Does your paper address subitem 18-i?

Copy and paste relevant sections from the manuscript (include quotes in quotation marks "like this" to indicate direct quotes from your manuscript), or elaborate on this item by providing additional information not in the ms, or briefly explain why the item is not applicable/relevant for your study

The manuscript does not include a subgroup analysis of comparing only users, as all analyses were conducted on the randomized sample, maintaining the integrity of the randomization process. No specific subset of participants (e.g., only those who completed all sessions or who engaged most actively with the intervention) was analyzed separately.

19) All important harms or unintended effects in each group
(for specific guidance see CONSORT for harms)

Does your paper address CONSORT subitem 19? *

Copy and paste relevant sections from the manuscript (include quotes in quotation marks "like this" to indicate direct quotes from your manuscript), or elaborate on this item by providing additional information not in the ms, or briefly explain why the item is not applicable/relevant for your study

"While VR-based BF was generally well-tolerated, some participants reported minor adverse events, including visual discomfort, difficulty adjusting the VR headset, mild dizziness, and occasional neck strain due to the weight of the equipment. A small number of participants experienced anxiety triggered by specific VR scenarios, such as height-related scenes. These events did not significantly impact the study's outcomes but highlight the need for ergonomic improvements to the VR headset and enhanced content design to minimize motion sickness and anxiety triggers. Addressing these issues in future iterations could improve participant comfort and engagement."

19-i) Include privacy breaches, technical problems

Include privacy breaches, technical problems. This does not only include physical “harm” to participants, but also incidents such as perceived or real privacy breaches [1], technical problems, and other unexpected/unintended incidents. “Unintended effects” also includes unintended positive effects [2].

	1	2	3	4	5	
subitem not at all important	○	○	○	○	◉	essential

선택해제

Does your paper address subitem 19-i?

Copy and paste relevant sections from the manuscript (include quotes in quotation marks "like this" to indicate direct quotes from your manuscript), or elaborate on this item by providing additional information not in the ms, or briefly explain why the item is not applicable/relevant for your study

"While VR-based BF was generally well-tolerated, some participants reported minor adverse events, including visual discomfort, difficulty adjusting the VR headset, mild dizziness, and occasional neck strain due to the weight of the equipment. A small number of participants experienced anxiety triggered by specific VR scenarios, such as height-related scenes. These events did not significantly impact the study's outcomes but highlight the need for ergonomic improvements to the VR headset and enhanced content design to minimize motion sickness and anxiety triggers. Addressing these issues in future iterations could improve participant comfort and engagement."

19-ii) Include qualitative feedback from participants or observations from staff/researchers

Include qualitative feedback from participants or observations from staff/researchers, if available, on strengths and shortcomings of the application, especially if they point to unintended/unexpected effects or uses. This includes (if available) reasons for why people did or did not use the application as intended by the developers.

	1	2	3	4	5	
subitem not at all important	○	○	○	◉	○	essential

선택해제

Does your paper address subitem 19-ii?

Copy and paste relevant sections from the manuscript (include quotes in quotation marks "like this" to indicate direct quotes from your manuscript), or elaborate on this item by providing additional information not in the ms, or briefly explain why the item is not applicable/relevant for your study

내 답변

DISCUSSION

22) Interpretation consistent with results, balancing benefits and harms, and considering other relevant evidence

NPT: In addition, take into account the choice of the comparator, lack of or partial blinding, and unequal expertise of care providers or centers in each group

22-i) Restate study questions and summarize the answers suggested by the data, starting with primary outcomes and process outcomes (use)

Restate study questions and summarize the answers suggested by the data, starting with primary outcomes and process outcomes (use).

	1	2	3	4	5	
subitem not at all important	○	○	○	○	◉	essential

선택해제

Does your paper address subitem 22-i? *

Copy and paste relevant sections from the manuscript (include quotes in quotation marks "like this" to indicate direct quotes from your manuscript), or elaborate on this item by providing additional information not in the ms, or briefly explain why the item is not applicable/relevant for your study

The study questions were whether VR-based biofeedback could improve sleep quality and alleviate depressive and anxiety symptoms in individuals with depressive and anxiety symptoms (DAS). Specifically, the primary focus was on evaluating the efficacy of VR-based BF in improving sleep quality, as measured by the PSQI, and determining its impact on depressive and anxiety symptoms compared to conventional BF. The primary outcomes demonstrated significant improvements in global PSQI scores, sleep latency, sleep disturbance, and daytime dysfunction in both the DAS/VR and DAS/BF groups, indicating that VR-based BF is effective in improving sleep quality.
The process outcomes were also notable, as VR technology enhanced participant engagement and facilitated adherence to the intervention. The HC/VR group exhibited measurable improvements in sleep quality, although less pronounced than in the DAS groups, suggesting that VR-based BF could benefit both clinical and non-clinical populations.

22-ii) Highlight unanswered new questions, suggest future research

Highlight unanswered new questions, suggest future research.

	1	2	3	4	5	
subitem not at all important	○	○	○	○	◉	essential

선택해제

Does your paper address subitem 22-ii?

Copy and paste relevant sections from the manuscript (include quotes in quotation marks "like this" to indicate direct quotes from your manuscript), or elaborate on this item by providing additional information not in the ms, or briefly explain why the item is not applicable/relevant for your study

내 답변

20) Trial limitations, addressing sources of potential bias, imprecision, and, if relevant, multiplicity of analyses

20-i) Typical limitations in ehealth trials

Typical limitations in ehealth trials: Participants in ehealth trials are rarely blinded. Ehealth trials often look at a multiplicity of outcomes, increasing risk for a Type I error. Discuss biases due to non-use of the intervention/usability issues, biases through informed consent procedures, unexpected events.

	1	2	3	4	5	
subitem not at all important	○	○	○	○	◉	essential

선택해제

Does your paper address subitem 20-i? *

Copy and paste relevant sections from the manuscript (include quotes in quotation marks "like this" to indicate direct quotes from your manuscript), or elaborate on this item by providing additional information not in the ms, or briefly explain why the item is not applicable/relevant for your study

First, the single-center design may limit the generalizability of the findings. Expanding the study to include multiple centers and diverse populations would provide a broader perspective on the efficacy of VR-based BF. "Second, this study is that participants were self-referred, responding voluntarily to recruitment advertisements."

21) Generalisability (external validity, applicability) of the trial findings

NPT: External validity of the trial findings according to the intervention, comparators, patients, and care providers or centers involved in the trial

21-i) Generalizability to other populations

Generalizability to other populations: In particular, discuss generalizability to a general Internet population, outside of a RCT setting, and general patient population, including applicability of the study results for other organizations

	1	2	3	4	5	
subitem not at all important	○	○	○	○	◉	essential

선택해제

Does your paper address subitem 21-i?

Copy and paste relevant sections from the manuscript (include quotes in quotation marks "like this" to indicate direct quotes from your manuscript), or elaborate on this item by providing additional information not in the ms, or briefly explain why the item is not applicable/relevant for your study

내 답변

21-ii) Discuss if there were elements in the RCT that would be different in a routine application setting

Discuss if there were elements in the RCT that would be different in a routine application setting (e.g., prompts/reminders, more human involvement, training sessions or other co-interventions) and what impact the omission of these elements could have on use, adoption, or outcomes if the intervention is applied outside of a RCT setting.

	1	2	3	4	5	
subitem not at all important	○	○	◉	○	○	essential

선택해제

Does your paper address subitem 21-ii?

Copy and paste relevant sections from the manuscript (include quotes in quotation marks "like this" to indicate direct quotes from your manuscript), or elaborate on this item by providing additional information not in the ms, or briefly explain why the item is not applicable/relevant for your study

In the RCT, a therapist guided participants through the VR-based biofeedback (BF) sessions and provided relaxation instructions. In routine practice, the level of human involvement might be reduced, potentially affecting the efficacy and user experience.

OTHER INFORMATION

23) Registration number and name of trial registry

Does your paper address CONSORT subitem 23? *

Copy and paste relevant sections from the manuscript (include quotes in quotation marks "like this" to indicate direct quotes from your manuscript), or elaborate on this item by providing additional information not in the ms, or briefly explain why the item is not applicable/relevant for your study

Trial Registration: Clinical Research Information Service (CRIS) KCT0004434; https://cris.nih.go.kr.

24) Where the full trial protocol can be accessed, if available

Does your paper address CONSORT subitem 24? *

Cite a Multimedia Appendix, other reference, or copy and paste relevant sections from the manuscript (include quotes in quotation marks "like this" to indicate direct quotes from your manuscript), or elaborate on this item by providing additional information not in the ms, or briefly explain why the item is not applicable/relevant for your study

key methodological details are outlined in the Methods section of the manuscript

25) Sources of funding and other support (such as supply of drugs), role of funders

Does your paper address CONSORT subitem 25? *

Copy and paste relevant sections from the manuscript (include quotes in quotation marks "like this" to indicate direct quotes from your manuscript), or elaborate on this item by providing additional information not in the ms, or briefly explain why the item is not applicable/relevant for your study

Funding sources had no role in study design, data collection, data analysis, data interpretation, writing of this paper, or the decision to submit this paper for publication.

X27) Conflicts of Interest (not a CONSORT item)

X27-i) State the relation of the study team towards the system being evaluated

In addition to the usual declaration of interests (financial or otherwise), also state the relation of the study team towards the system being evaluated, i.e., state if the authors/evaluators are distinct from or identical with the developers/sponsors of the intervention.

	1	2	3	4	5	
subitem not at all important	○	○	○	○	◉	essential

선택해제

Does your paper address subitem X27-i?

Copy and paste relevant sections from the manuscript (include quotes in quotation marks "like this" to indicate direct quotes from your manuscript), or elaborate on this item by providing additional information not in the ms, or briefly explain why the item is not applicable/relevant for your study

내 답변

About the CONSORT EHEALTH checklist

As a result of using this checklist, did you make changes in your manuscript? *

- ○ yes, major changes
- ◉ yes, minor changes
- ○ no

What were the most important changes you made as a result of using this checklist?

내 답변

How much time did you spend on going through the checklist INCLUDING making changes in your manuscript *

I dedicated approximately four days to carefully address each subitem, ensure accuracy and compliance with the guidelines.

As a result of using this checklist, do you think your manuscript has improved? *

- (x) yes
- ( ) no
- ( ) 기타:

Would you like to become involved in the CONSORT EHEALTH group?

This would involve for example becoming involved in participating in a workshop and writing an "Explanation and Elaboration" document

- ( ) yes
- ( ) no
- ( ) 기타:

Any other comments or questions on CONSORT EHEALTH

내 답변

STOP - Save this form as PDF before you click submit

To generate a record that you filled in this form, we recommend to generate a PDF of this page (on a Mac, simply select "print" and then select "print as PDF") before you submit it.

When you submit your (revised) paper to JMIR, please upload the PDF as supplementary file.

Don't worry if some text in the textboxes is cut off, as we still have the complete information in our database. Thank you!

Final step: Click submit !

Click submit so we have your answers in our database!

제출 양식 지우기

Google Forms를 통해 비밀번호를 제출하지 마세요.

이 콘텐츠는 Google이 만들거나 승인하지 않았습니다. - 서비스 약관 - 개인정보처리방침

양식이 의심스러운가요? 보고서

Google 설문지
